# Comparative analysis of the complete chloroplast genomes from six Neotropical species of Myrteae (Myrtaceae)

**DOI:** 10.1590/1678-4685-GMB-2019-0302

**Published:** 2020-05-08

**Authors:** Nureyev F. Rodrigues, Natalia Balbinott, Igor Paim, Frank Guzman, Rogerio Margis

**Affiliations:** 1 Universidade Federal do Rio Grande do Sul, Departamento de Biofísica, Laboratório de Genomas e Populações de Plantas, Porto Alegre, RS, Brazil.; 2 Universidade Federal do Rio Grande do Sul , Centro de Biotecnologia, PPGBCM, Porto Alegre, RS, Brazil; 3 Universidade Federal do Rio Grande do Sul, Departamento de Biofísica, Porto Alegre, RS, Brazil.

**Keywords:** cpDNA, genomic resource, populational genetics, plastid, conservation

## Abstract

Myrteae is the largest and most diverse tribe within Myrtaceae and represents the majority of its diversity in the Neotropics. Members of Myrteae hold ecological importance in tropical biomes for the provision of food sources for many animal species. Thus, due to its several roles, a growing interest has been addressed to this group. In this study, we report the sequencing and *de novo* assembly of the complete chloroplast (cp) genomes of six Myrteae species: *Eugenia brasiliensis, E. pyriformis, E. nitida, Myrcianthes pungens, Plinia edulis* and *Psidium cattleianum.* We characterized genome structure, gene content, and identified SSRs to detect variation within Neotropical Myrteae. The six newly sequenced plastomes exhibit a typical quadripartite structure, gene content and organization highly conserved among Myrtaceae species. Some differences in genome length, protein-coding genes and non-coding regions were found. Besides, IR boundaries present structural changes among species. Increased sequence diversity was observed in some intergenic regions, suggesting their suitability for investigating intraand interspecific genetic diversity in populational studies. These data also contribute to the improvement of taxa sampling in further phylogenetic investigations to understand Myrtaceae evolution.

Myrtaceae encompasses over 6000 species of shrubs and trees, classified in 144 genera and subdivided into 17 tribes (Wilson *et al*., 2005; WCSP, 2019). This angiosperm family has a predominant Southern-Hemisphere distribution and is assumed to be of Gondwanan origin, being an important component in the forests of Southeast Asia, Australia, and South America (Wilson *et al*., 2005; Thornhill *et al*., 2015). In the Neotropical region, most of Myrtaceae is represented by the tribe Myrteae, which comprises over 50 genera and 2500 species, representing half of the diversity of the family (Wilson *et al*., 2005; WCSP, 2019). Myrteae species play an important ecological role in Neotropical environments as foraging resources to animals, especially to a variety of bee species (Fidalgo and Kleinert, 2009). Besides that, some studies focused on specific classes of compounds produced by Myrtaceae, such as terpenes, which present commercial uses (Guzman *et al*., 2014). Other studies have demonstrated the antifungal, antioxidant, antiinflammatory, gastroprotective, and other bioactivities of

Myrteae species from Brazil (Salvador *et al*., 2011; Souza Moreira *et al*., 2019). Thus, due to its plethora of roles, a growing interest has been addressed to this group as a model for evolutionary, ecological and applied studies. For this study, leaves from *Eugenia brasiliensis, Eugenia pyriformis, Eugenia nitida, Myrcianthes pungens, Plinia edulis* and *Psidium cattleianum* trees were from a private *in vivo* collection in Gravataí, RS, Brazil (latitude (S): 29°51’52"; longitude (W): 50°53’53") and used to isolate chloroplasts by the modified high salt method, followed by cpDNA extraction with the CTAB method (Vieira *et al*., 2014). DNA quality was evaluated by electrophoresis in a 1% agarose gel, and DNA quantity was determined using a NanoDrop spectrophotometer (NanoDrop Technologies, Wilmington, DE, USA). For each species, one genomic paired-end library of 150 nt length was generated using an Illumina HiSeq2000 platform (Macrogen). The bases with quality below Q30 and adapter contamination were trimmed using Trim Galore!0.4.2, with a 50 bases minimum allowed. Paired-end sequence reads were filtered against 28 Myrtaceae plastomes (Table S1) using BWA software (Li and Durbin, 2009) with two mismatches allowed. Mapped reads were used for the *de novo* assembly with ABySS software (Jackman *et al*., 2017). The plastome scaffolds were orientated by MUMmer (Delcher *et al*., 2003) using *Eugenia uniflora* (NC_026450.1), *Plinia trunciflora* (NC_034801.1) or *Psidium guajava* (NC_033355.1) as reference genomes for species of the same or closer genus. Genes were annotated using GeSeq (Tillich *et al*., 2017) by BLAST searches with 80% similarity. Circular plastome maps were drawn using the OGDRAW web toolkit (Lohse *et al*., 2013). IRa and IRb boundaries were analyzed using IRScope (Amiryousefi *et al*., 2018). For each species, local mVISTA (Frazer *et al*., 2004) was used to pairwise align plastomes with their respective reference. An overall genome comparison was performed with BLAST Ring Image Generator (BRIG) (Alikhan *et al*., 2011). Krait v0.11.4 (Du *et al*., 2018) was used to search and annotate perfect SSRs using the genomes and their annotated GFF3 file. The parameters for minimum repeat numbers were 8, 4, 3, 3, 3, 3 for mono-, di-, tri-, tetra-, penta- and hexanucleotide SSRs, respectively. DNA sequencing libraries were produced for each species, and these comprised 34.1-48.7 M raw Illumina paired-end reads (summarized in Table S2). The percentage of removed reads due to trimming ranged from 1.17-1.45%. The number of filtered reads was 1.1-2.2 M reads. The obtained reads were *de novo* assembled into scaffolds that completely covered each plastome, without any gaps. The number of assembled scaffolds obtained ranged from four to eight. The minimum coverage ranged from 13 to 46 reads and the maximum coverage, 1,508-3,620 reads. The complete sequences were submitted to GenBank at accession numbers MN095407 to MN095411 and MN095413 (Table 1). The complete plastomes of six Myrteae have a narrow size range, from 157,683 bp in *E. nitida* to 159,631 bp in *P. edulis,* similar to the size of Myrtaceae species plastomes (Eguiluz *et al*., 2017 a,b; Machado *et al*., 2017). Figures S1, S2, S3, S4, S5, S6 present the genome maps for each species. Four well-defined regions are present in all newly assembled genomes. Inverted regions (IR) ranged from ~26.3 to 26.4 kbp and had the smallest size variation, up to 78 bp. Short single copy (SSC) sections have ~18.2-18.5 kbp, while long single copy (LSC) sections have ~86.4-88.2 kbp (Table 1). Protein coding sequences comprise ~50% of the genome, rRNAs and tRNAs comprise ~7%, and non-coding regions, such as introns, pseudogenes and intergenic spacers correspond to ~43% (Table 1). Genome structure analysis showed a high degree of synteny among evaluated species (Figure 1). Genomes contained 129 genes in total, corresponding to 78 single-copy protein-coding genes, 30 transfer RNA (tRNA) genes, four ribosomal genes (rRNA) and one pseudogene (*ycf1*) (Figure 1, Table 1). In general, genomic features, such as size, structure, and gene abundance are similar to previously described Myrtaceae species (Eguiluz *et al*., 2017a,b). Despite the similarity in genomic features, the mVISTA comparison against each respective reference genome showed that some regions display lower similarity (Figures S7, S8, S9). Non-coding regions, particularly the intergenic, had lower conservation and, therefore, more variation, such as *psbI-trnS, trnT-psbD, trnS-psbZ-trnG, accD-psaI,* and *ndhF-rp132* in *Eugenia* and *Myrcianthes; trnS-trnR, atpF-atpH, trnT-trnL,* and *rpl32-trnL* in *Plinia*; and most intergenic regions of LSC in *Psidium.* Regarding protein-coding genes, we observed a conservation decrease in *accD* and *ccsA* in *Eugenia* species and *M. pungens*; and *rpoC2* in *Plinia* and *Psidium.* Protein-coding genes *matK, ndhF* and *ycf1*, showed more nucleotide diversity (4.6 to 6.1%) in all analyzed species (Figures S7, S8, S9, S10). This diversity corroborates previous studies based on plastidial genes and non-coding regions with contrasting substitution rates (Thornhill *et al*., 2015; Machado *et al*., 2017). Some structural changes in the IRa and IRb boundaries were found for the evaluated species (Figure 2). Within the IRb-LSC boundaries, the boundaries of the *rps19* gene were located on the left side. In the IRb region, except for *M. pungens,* the IRb-LSC boundary was embedded in *rps19* and had a length of three bp in *Eugenia* (Figure 2A), ~30 bp in *Plinia* (Figure 2B), and 31 bp in *Psidium,* contained in the IRb (Figure 2C). The IRb-SSC boundaries were embedded in the *ycf1* pseudogene, ranging from one to eight bp in *Eugenia/Myrcianthes,* one and two bp in *Plinia,* and only one bp in *Psidium* species. The *ndhF* gene was located on the right side of the IRb-SSC at a distance from the boundary of 10 bp in *E. uniflora,* 36 to 121 bp in other *Eugenia,* 72 bp in *M. pungens,* 109 bp and 120 bp in *Plinia,* and 111 bp and 124 bp in *Psidium*. The SSC-IRa boundary was embedded in *ycf1,* with a length of 1047 to 1080 bp in *Eugenia/Myrcianthes,* 1080 and 1011 bp in *Plinia,* and 1071 and 1079 bp in *Psidium* in the IRa region. The *trnH-GUG* gene was located on the right side of the IRa-LSC boundary ranging from 11 to 52 bp in *Eugenia/Myrcianthes,* from 3 to 10 bp in *Plinia* and 10 to 14 bp in *Psidium.* The contraction and expansion of IR regions are measurable events of plastome evolution. These results demonstrate a genus-specific IR conservation, which can be considered one of the reasons for genome size variation among species. In this work, we present, for the first time, a characterization of IR boundaries from species of the same genus in Myrteae because they compared different genera, previous studies could not report a significant variation in IRb-SSC border within Myrteae species (Eguiluz *et al*., 2017a,b; Machado *et al*., 2017). All plastomes presented a similar number of SSRs. In total, over 315 SSRs were identified for each species (Table 2). The mononucleotide SSRs of A/T were the most frequent, varying in number from 85/98 in *E. brasiliensis/E. pyriformis* to 93/103 in *P. edulis/P. cattleianum* (Table S3). This AT richness was already demonstrated in previous studies and reflects the lower GC content in these plastid genomes (Eguiluz *et al*., 2017a,b). The secondmost common were the trinucleotide SSRs, ranging in number from 61 in *E. nitida* to 71 in *P. edulis.* In addition, the number of SSRs located in different regions were similar: in intergenic regions ranging from 171 in *E. nitida* to 185 in *P. edulis*; in genes, ranging from 96 in *E. brasiliensis, E. pyriformis, M. pungens,* and *P. cattleianum,* and 98 in *E. nitida* and *P. edulis*; and in introns, ranging from 43 in *E. brasiliensis* to 47 in *E. nitida, P. edulis,* and *P. cattleianum* (Table 2). Hexanucleotide SSRs could not be found in the genomes. All found SRRs are listed in Table S4. These SSR results provide more information on molecular markers that could be used to evaluate intra- and interspecific diversity. This work provides reference genomes for six Neotropical Myrtaceae species, increasing the genetic information available for the Myrteae tribe, and allowing the improvement of taxa sampling in further investigations into Myrtaceae evolution.

**Table 1:** Myrteae chloroplast genome features.

Feature	*Eugenia* *brasiliensis*	*Eugenia nitida*	*Eugenia* *pyriformis*	*Myrcianthes* *pungens*	*Plinia edulis*	*Psidium* *cattleianum*
GenBank accession	MN095407	MN095411	MN095410	MN095409	MN095413	MN095408
Total cpDNA size (bp)	158,251	157,683	158,569	159,239	159,631	159,088
LSC size (bp)	87,201	86,436	87,189	87,910	88,202	87,798
SSC size (bp)	18,290	18,349	18,566	18,587	18,579	18,512
IR size (bp)	26,380	26,449	26,407	26,371	26,425	26,390
Protein coding regions (%)	50.01	50.22	49.90	49.67	49.48	49.65
rRNA and tRNA (%)	7.48	7.51	7.46	7.43	7.43	7.44
Introns size (% total)	13.02	13.09	13.02	12.97	12.90	12.92
Intergenic sequences (%)	31.21	30.85	31.32	31.63	31.79	31.58
Number of genes	129	129	129	129	129	129
Number of different protein coding genes	78	78	78	78	78	78
Number of different tRNA genes	30	30	30	30	30	30
Number of different rRNA genes	4	4	4	4	4	4
Number of duplicated genes	16	16	16	16	16	16
Pseudogenes	1	1	1	1	1	1
GC content (%)	36.95	37.04	37.01	36.94	36.93	37.05

**Figure 1:**
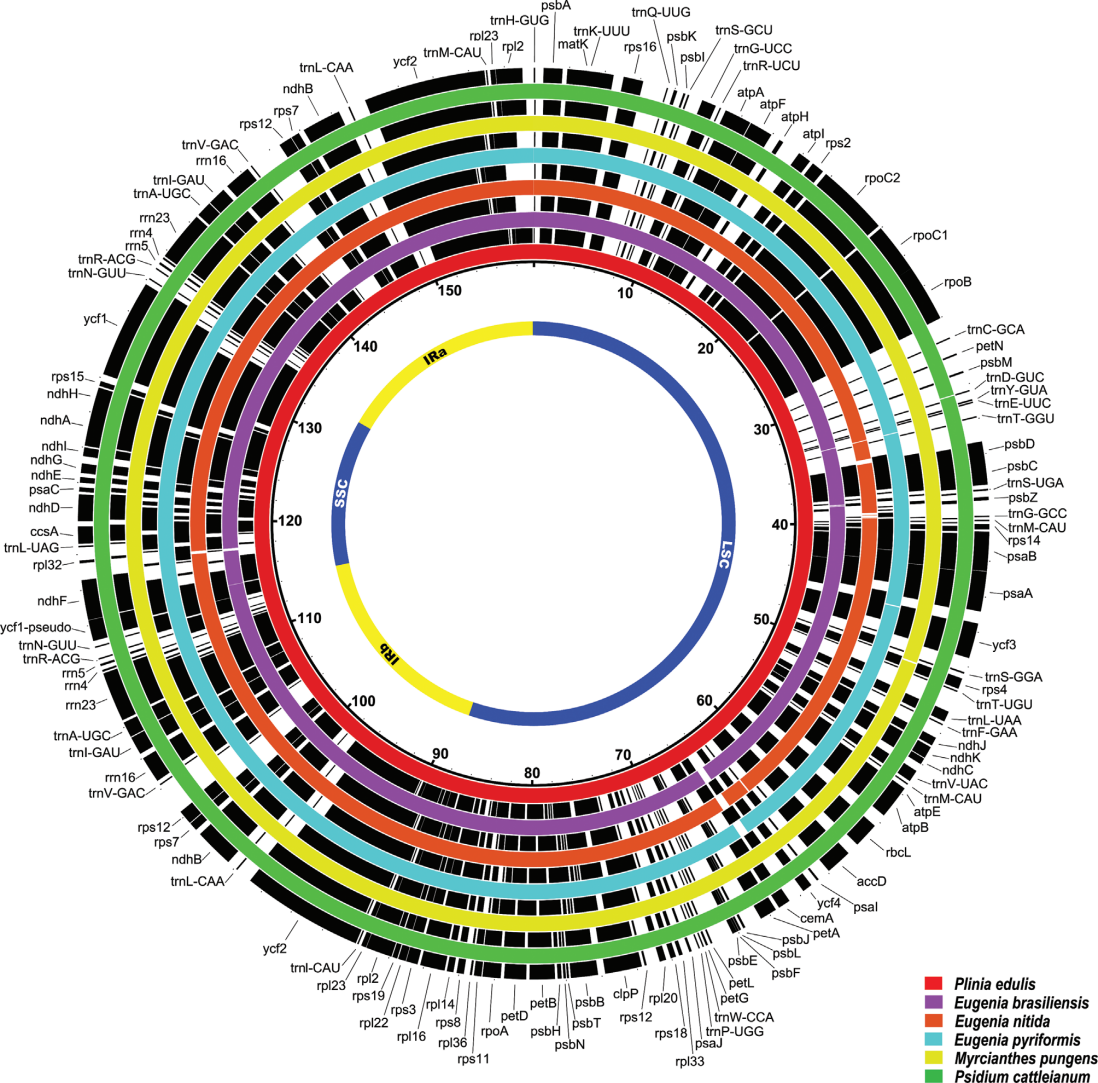
Complete gene map of six Myrteae plastomes. Gene annotations are in black. The plastomes are in red *(P. edulis),* purple *(E. brasiliensis),* orange *(E. nitida),* blue (E. pyriformis), yellow *(M. pungens),* green (P. *cattleianum*). LSC: large single-copy region; SSC: small single-copy region; IR: inverted repeat. The numbers near *P. edulis* (red circle) represent the nucleotide positions (in kbp).

**Figure 2:**
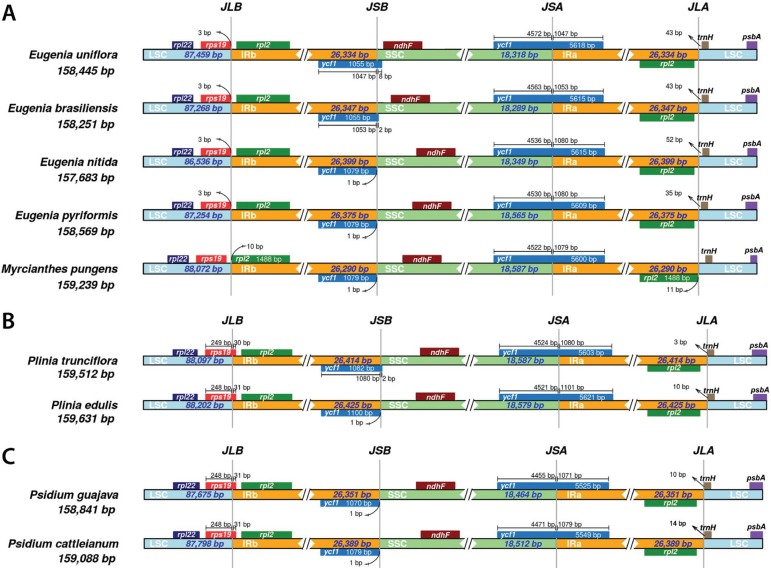
Comparison ofborder positions ofLSC, SSC and IRamong (A) *Eugenia uniflora,* (B) *Psidium guajava* and (C) *Plinia trunciflora* andrelated new species. JSA/JSB, junction of SSC-IRa/IRb; JLA/JLB, junction of LSC-IRa/IRb. Boxes above or under the main line indicate the predicted genes; *ycfl* pseudogenes are at JSB and their lengths are displayed in the corresponding regions. The figure is not to scaled, and shows relative changes at or near the IR-SC borders.

**Table 2:** Types, locations, and numbers of SSRs in the chloroplast genomes of six Myrteae species.

Feature		*Eugenia brasiliensis*	*Eugenia nitida*	*Eugenia pyriformis*	*Myrcianthes punges*	*Plinia edulis*	*Psidium cattleianum*
SSR type	Mono	188	193	189	194	197	198
	Di	50	49	49	52	49	45
	Tri	65	61	65	63	71	64
	Tetra	12	12	13	13	13	13
	Penta	0	1	1	0	0	1
Location	Intergenic	176	171	175	181	185	178
	Genes	96	98	96	96	98	96
	Introns	43	47	46	45	47	47
	Total	315	316	317	322	330	321

## Supplementary material

The following online material is available for this article:

Figure S1 - Gene map of *Eugenia brasiliensis* chloroplast genome.

Figure S2 - Gene map of *Eugenia nitida* chloroplast genome.

Figure S3 - Gene map of *Eugenia pyriformis* chloroplast genome.

Figure S4 - Gene map of *Myrcianthes pungens* chloroplast genome.

Figure S5 - Gene map of *Plinia edulis* chloroplast genome.

Figure S6 - Gene map of *Psidium cattleianum* chloroplast genome.

Figure S7 - Sequence identity plot comparing plastomes of *Myrcianthes* and *Eugenia* species.

Figure S8 - Sequence identity plot comparing plastomes of *Plinia* species.

Figure S9 - Sequence identity plot comparing plastomes of *Psidium* species.

Figure S10 - Nucleotide alignment of *accD, ccsA, rpoC2, matK, ndhF* and *ycfl*.

Table S1 - List of 28 Myrtaceae chloroplast genomes.

Table S2 - Summary of libraries and assemblies from the chloroplast genomes.

Table S3 - List of simple sequence repeats of six Myrteae plastomes.

Table S4 - List of simple sequence repeats with the respective position in plastome.
