# Supplementary material for: Comparative analysis of the complete chloroplast genomes from six Neotropical species of Myrteae (Myrtaceae)
Source: Genet Mol Biol. 2020 May 8;43(2):e20190302. doi: 10.1590/1678-4685-GMB-2019-0302 (PMC7212760; doi:10.1590/1678-4685-GMB-2019-0302)
Supplement: Supplementary file 1 [file 1415-4757-GMB-43-2-e20190302-s11.pdf]

Supplementary Material to "Comparative analysis of the complete chloroplast genomes from six Neotropical species of Myrteae (Myrtaceae)"

Table S1 - List of 28 Myrtaceae chloroplast genomes used in chloroplast genome assembling of six Myrteae species.

**No.**	**Taxon**	**GenBank accession number**	**Study**
1	KX289887.1	*Acca sellowiana*	(Machado *et al.* 2017)
2	MG925369.1	*Eucalyptus grandis*	(Pinard *et al.* 2019)
3	NC_008115.1	*Eucalyptus globulus* subsp. *globulus*	(Steane 2005)
4	NC_022395.1	*Eucalyptus nitens*	(Bayly *et al.* 2013)
5	NC_022396.1	*Eucalyptus aromaphloia*	(Bayly *et al.* 2013)
6	NC_022397.1	*Eucalyptus saligna*	(Bayly *et al.* 2013)
7	NC_022398.1	*Eucalyptus camaldulensis*	(Bayly *et al.* 2013)
8	NC_022399.1	*Eucalyptus deglupta*	(Bayly *et al.* 2013)
9	NC_022400.1	*Eucalyptus spathulata*	(Bayly *et al.* 2013)
10	NC_022401.1	*Eucalyptus torquata*	(Bayly *et al.* 2013)
11	NC_022402.1	*Eucalyptus diversicolor*	(Bayly *et al.* 2013)
12	NC_022403.1	*Eucalyptus salmonophloia*	(Bayly *et al.* 2013)
13	NC_022404.1	*Eucalyptus microcorys*	(Bayly *et al.* 2013)
14	NC_022405.1	*Eucalyptus guilfoylei*	(Bayly *et al.* 2013)
15	NC_022406.1	*Eucalyptus erythrocorys*	(Bayly *et al.* 2013)
16	NC_022407.1	*Corymbia gummifera*	(Bayly *et al.* 2013)
17	NC_022408.1	*Corymbia maculata*	(Bayly *et al.* 2013)
18	NC_022409.1	*Corymbia eximia*	(Bayly *et al.* 2013)
19	NC_022410.1	*Corymbia tessellaris*	(Bayly *et al.* 2013)
20	NC_022411.1	*Angophora floribunda*	(Bayly *et al.* 2013)
21	NC_022412.1	*Angophora costata*	(Bayly *et al.* 2013)
22	NC_022413.1	*Allosyncarpia ternata*	(Bayly *et al.* 2013)
23	NC_022414.1	*Stockwellia quadrifida*	(Bayly *et al.* 2013)
24	NC_027744.1	*Eugenia uniflora*	(Eguiluz *et al.* 2017a)
25	NC_033355.1	*Psidium guajava*	Unpublished
26	NC_034801.1	*Plinia trunciflora*	(Eguiluz *et al.* 2017b)
27	NC_039395.1	*Plinia cauliflora*	Unpublished
28	NC_039557.1	*Plinia aureana*	Unpublished

References

Bayly MJ, Rigault P, Spokevicius A, Ladiges PY, Ades PK, Anderson C, Bossinger G, Merchant A, Udovicic F, Woodrow IE *et al.* (2013) Chloroplast genome analysis of Australian eucalypts – Eucalyptus, Corymbia, Angophora, Allosyncarpia and Stockwellia (Myrtaceae). Mol Phylogenet Evol 69:704–716.

Eguiluz M, Rodrigues NF, Guzman F, Yuyama P and Margis R (2017a) The chloroplast genome sequence from Eugenia uniflora, a Myrtaceae from Neotropics. Plant Syst Evol 303:1199–1212.

Eguiluz M, Yuyama PM, Guzman F, Rodrigues NF and Margis R (2017b) Complete sequence and comparative analysis of the chloroplast genome of Plinia trunciflora. Genet Mol Biol 40:871–876.

Machado L de O, Vieira L do N, Stefenon VM, Oliveira Pedrosa F de, Souza EM de, Guerra MP and Nodari RO (2017) Phylogenomic relationship of feijoa (Acca sellowiana (O.Berg) Burret) with other Myrtaceae based on complete chloroplast genome sequences. Genetica 145:163–174.

Pinard D, Myburg AA and Mizrachi E (2019) The plastid and mitochondrial genomes of Eucalyptus grandis. BMC Genomics 20:132.

Steane DA (2005) Complete Nucleotide Sequence of the Chloroplast Genome from the Tasmanian Blue Gum, Eucalyptus globulus (Myrtaceae). DNA Res 12:215–220.
