# Supplementary material for: Comparative analysis of the complete chloroplast genomes from six Neotropical species of Myrteae (Myrtaceae)
Source: Genet Mol Biol. 2020 May 8;43(2):e20190302. doi: 10.1590/1678-4685-GMB-2019-0302 (PMC7212760; doi:10.1590/1678-4685-GMB-2019-0302)
Supplement: Supplementary file 2 [file 1415-4757-GMB-43-2-e20190302-s12.pdf]

Supplementary Material to "Comparative analysis of the complete chloroplast genomes from six Neotropical species of Myrteae (Myrtaceae)"

Table S2 – Summary of libraries and assemblies from chloroplast genome of six Myrteae species.

Feature	*E. brasiliensis*	*E. nitida*	*E. pyriformis*	*M. pungens*	*P. edulis*	*P. cattleianum*
Raw reads	39,271,776	46,914,730	34,174,022	48,777,706	36,919,420	38,628,416
Processed reads	38,751,186	46,233,438	33,718,411	48,186,086	36,486,495	38,158,315
Removed reads (%)	1.32%	1.45%	1.33%	1.21%	1.17%	1.21%
Filtered reads	2,228,859	2,229,459	1,303,789	1,402,123	1,565,949	1,184,911
Coverage (%)	100%	100%	100%	100%	100%	100%
Mean coverage	1,769	1,772	1,036	1,108	1,234	936
Minimum coverage	46	32	17	30	13	12
Maximum coverage	2,664	3,620	1,591	1,783	1,944	1,508
Assembled scaffolds	5	7	4	8	8	7
Gaps	0	0	0	0	0	0
