# Supplementary material for: Comparative analysis of the complete chloroplast genomes from six Neotropical species of Myrteae (Myrtaceae)
Source: Genet Mol Biol. 2020 May 8;43(2):e20190302. doi: 10.1590/1678-4685-GMB-2019-0302 (PMC7212760; doi:10.1590/1678-4685-GMB-2019-0302)
Supplement: Supplementary file 5 [file 1415-4757-GMB-43-2-e20190302-s3.pdf]

Supplementary Material to "Comparative analysis of the complete chloroplast genomes from six Neotropical species of Myrteae (Myrtaceae)"

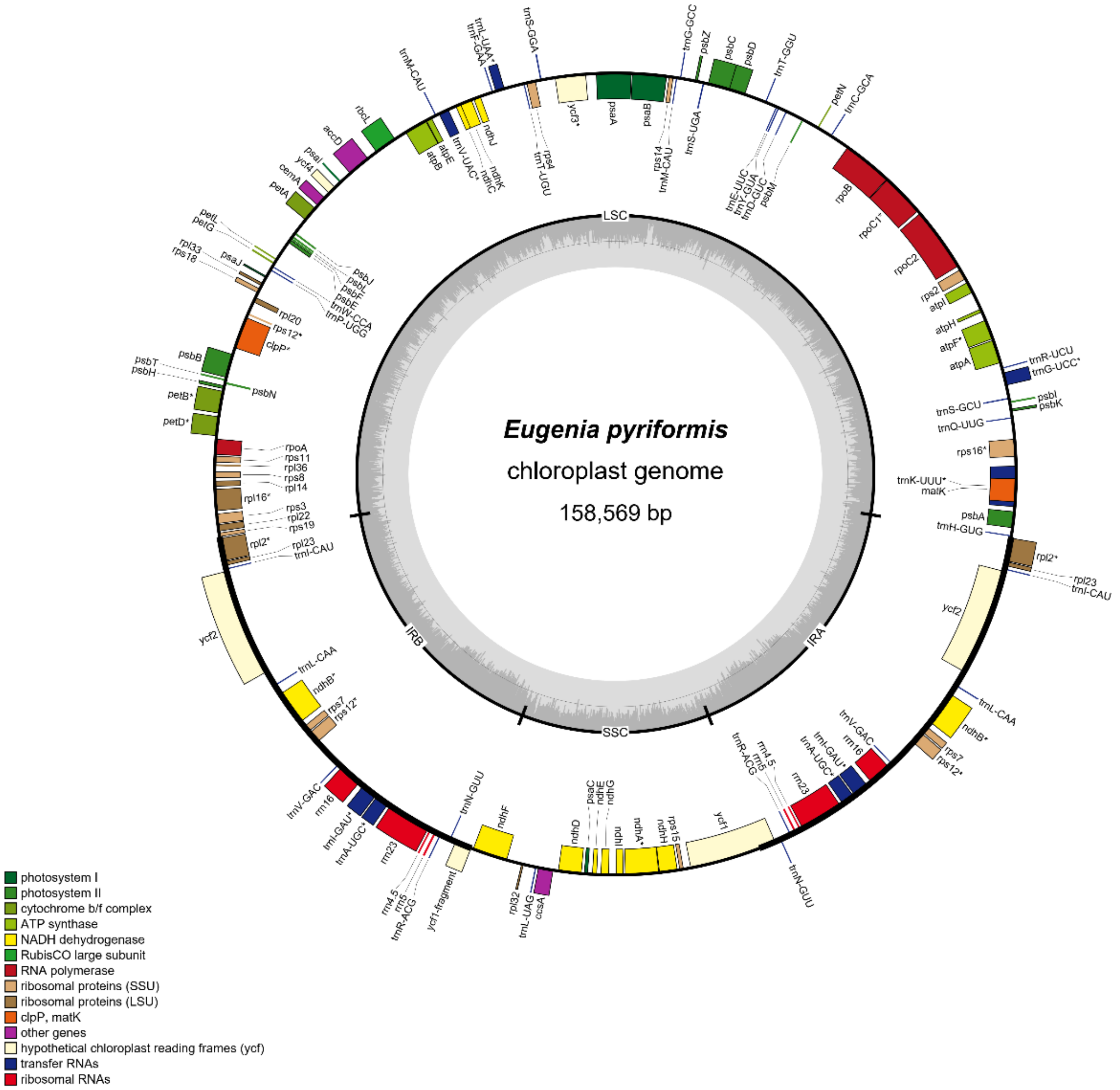

Figure S3 - Gene map of *Eugenia pyriformis* chloroplast genome.
