# Supplementary material for: Comparative analysis of the complete chloroplast genomes from six Neotropical species of Myrteae (Myrtaceae)
Source: Genet Mol Biol. 2020 May 8;43(2):e20190302. doi: 10.1590/1678-4685-GMB-2019-0302 (PMC7212760; doi:10.1590/1678-4685-GMB-2019-0302)
Supplement: Supplementary file 12 [file 1415-4757-GMB-43-2-e20190302-s10.pdf]

Supplementary Material to " Comparative analysis of the complete chloroplast genomes from six Neotropical species of Myrteae (Myrtaceae)"

accD

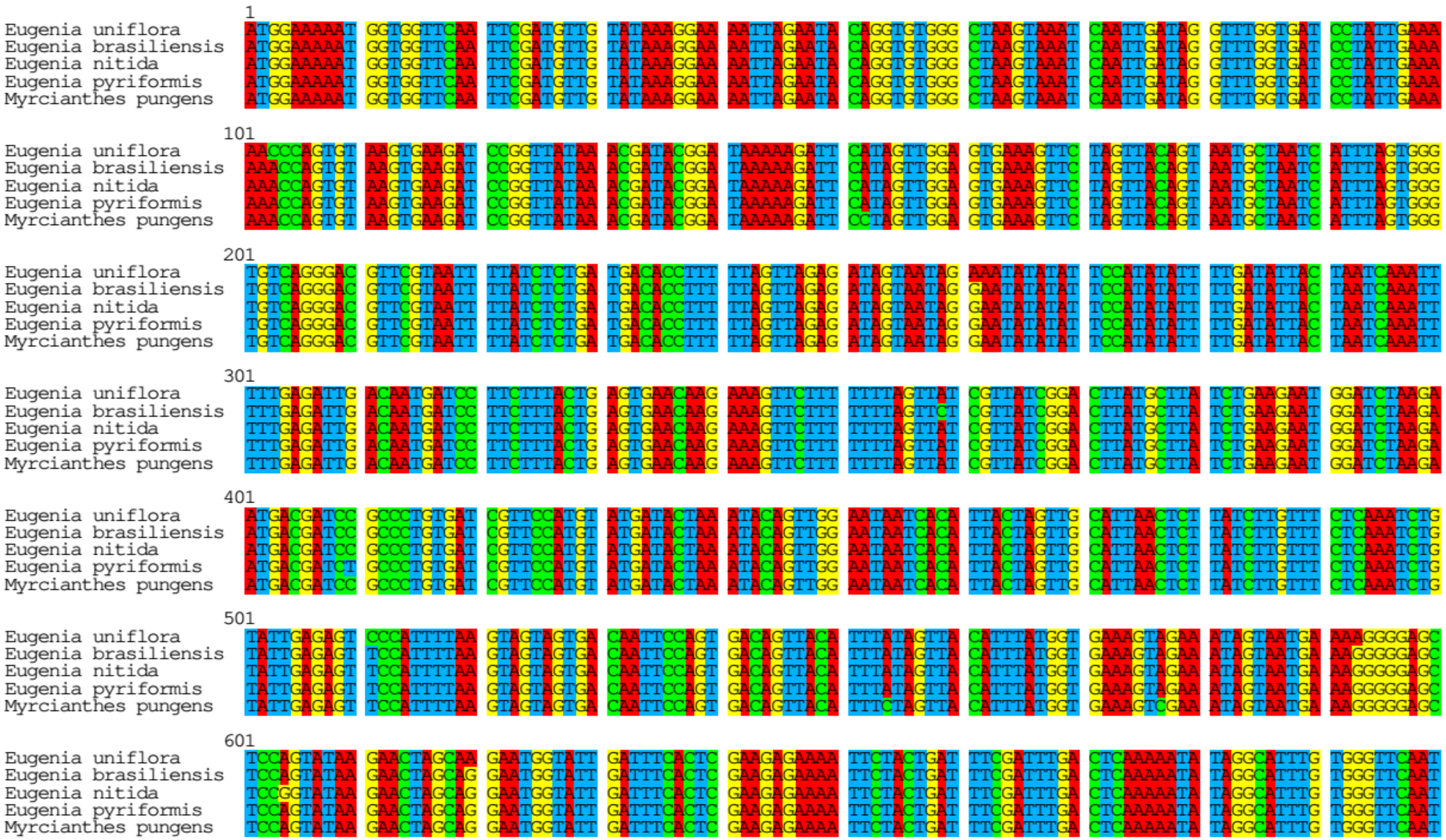

Figure S10 - Nucleotide alignment of *accD, ccsA, rpoC2, matK, ndhF* and *ycf1*.
