# Supplementary material for: Comparative analysis of the complete chloroplast genomes from six Neotropical species of Myrteae (Myrtaceae)
Source: Genet Mol Biol. 2020 May 8;43(2):e20190302. doi: 10.1590/1678-4685-GMB-2019-0302 (PMC7212760; doi:10.1590/1678-4685-GMB-2019-0302)
Supplement: Supplementary file 13 [file 1415-4757-GMB-43-2-e20190302-s13.pdf]

Supplementary Material to "Comparative analysis of the complete chloroplast genomes from six Neotropical species of Myrteae (Myrtaceae)"

Table S3 – List of simple sequence repeats present in the six Myrteae plastomes.

SSR	*Eugenia brasiliensis*	*Eugenia nitida*	*Eugenia pyriformis*	*Myrcianthes pungens*	*Plinia edulis*	*Psidium cattleianum*
A	85	88	87	89	93	93
C	3	4	3	3	2	1
G	2	1	1	1	1	1
T	98	100	98	101	101	103
AG	2	2	2	2	2	2
AT	21	21	20	20	20	18
CA	1	1	1	1	0	0
CT	3	3	3	3	4	3
GA	6	6	6	7	6	6
GT	0	1	0	0	0	0
TA	11	9	11	11	9	10
TC	6	6	6	8	8	6
AAC	2	2	2	2	2	2
AAG	2	2	2	2	4	2
AAT	4	3	3	3	4	4
ACC	1	1	1	1	1	1
AGA	4	4	4	4	4	4
AGT	1	1	2	1	1	1
ATA	4	4	4	4	4	3
ATC	1	1	1	1	1	1

SSR	*Eugenia brasiliensis*	*Eugenia nitida*	*Eugenia pyriformis*	*Myrcianthes pungens*	*Plinia edulis*	*Psidium cattleianum*
ATG	1	1	1	1	2	2
ATT	4	4	5	6	6	4
CAA	1	1	1	1	1	1
CAG	2	2	2	2	2	2
CTA	0	0	0	0	1	1
CTG	1	1	1	1	1	1
CTT	5	4	4	4	4	4
GAA	3	2	3	3	4	3
GCT	1	1	1	1	1	1
GGA	1	1	1	1	1	1
GGT	1	1	1	1	1	1
TAA	4	3	3	3	5	4
TAG	1	1	1	1	1	1
TAT	1	0	1	1	2	2
TCC	1	1	1	1	1	1
TCT	4	4	4	4	4	4
TGC	1	1	1	1	1	1
TTA	6	7	7	5	4	3
TTC	5	5	5	5	5	6
TTG	3	3	3	3	3	3
AATA	1	1	1	1	1	1
AGAT	1	1	1	1	1	0
ATAA	0	1	1	1	1	1
ATAG	1	1	1	1	1	1
ATTA	2	1	2	2	1	2
ATTT	1	1	1	1	1	1

SSR	*Eugenia brasiliensis*	*Eugenia nitida*	*Eugenia pyriformis*	*Myrcianthes pungens*	*Plinia edulis*	*Psidium cattleianum*
CTTG	1	1	1	1	1	1
GGTA	0	0	0	0	1	0
TAAA	1	0	0	0	0	0
TAAG	1	1	1	1	1	1
TAAT	1	1	1	1	1	1
TCTT	1	1	1	1	1	1
TTAT	0	0	0	0	0	1
TTTC	1	2	2	2	2	2
AATGG	0	1	0	0	0	1
TTTAT	0	0	1	0	0	0
**Total**	**315**	**316**	**317**	**322**	**330**	**321**
