# Supplementary material for: Comparative analysis of the complete chloroplast genomes from six Neotropical species of Myrteae (Myrtaceae)
Source: Genet Mol Biol. 2020 May 8;43(2):e20190302. doi: 10.1590/1678-4685-GMB-2019-0302 (PMC7212760; doi:10.1590/1678-4685-GMB-2019-0302)
Supplement: Supplementary file 14 [file 1415-4757-GMB-43-2-e20190302-s14.pdf]

Supplementary Material to "Comparative analysis of the complete chloroplast genomes from six Neotropical species of Myrteae (Myrtaceae)"

Table S4 – List of simple sequence repeats with respective position in plastome by species.

Species	Id	Motif	Type	Repeat	Start	End	Length
Eugenia brasiliensis	1	A	1	9	17	25	9
	2	T	1	9	141	149	9
	3	A	1	10	297	306	10
	4	CAG	3	4	1163	1174	12
	5	T	1	8	2146	2153	8
	6	TCC	3	3	2322	2330	9
	7	T	1	9	2457	2465	9
	8	CA	2	4	3076	3083	8
	9	T	1	11	4265	4275	11
	10	A	1	10	4504	4513	10
	11	A	1	11	4679	4689	11
	12	A	1	9	4741	4749	9
	13	TA	2	4	4790	4797	8
	14	T	1	9	4802	4810	9
	15	AGAT	4	3	4854	4865	12
	16	A	1	8	5480	5487	8
	17	A	1	8	5645	5652	8
	18	A	1	8	6511	6518	8
	19	A	1	8	6668	6675	8
	20	A	1	8	6737	6744	8
	21	TA	2	4	7472	7479	8
	22	A	1	10	7900	7909	10
	23	A	1	11	8137	8147	11
	24	T	1	8	8300	8307	8
	25	T	1	10	8429	8438	10
	26	A	1	12	8440	8451	12
	27	A	1	10	8690	8699	10
	28	A	1	11	8716	8726	11
	29	A	1	9	8889	8897	9
	30	A	1	8	9023	9030	8
	31	T	1	9	9067	9075	9
	32	TAT	3	3	9268	9276	9
	33	A	1	10	9316	9325	10
	34	T	1	10	9539	9548	10
	35	T	1	9	9856	9864	9
	36	AT	2	4	10493	10500	8
	37	A	1	8	10671	10678	8
	38	A	1	14	10686	10699	14
	39	T	1	9	10915	10923	9
	40	A	1	9	10987	10995	9

Species	Id	Motif	Type	Repeat	Start	End	Length
	41	ATTA	4	3	11006	11017	12
	42	ATTT	4	3	11054	11065	12
	43	A	1	9	12620	12628	9
	44	T	1	10	13179	13188	10
	45	A	1	9	13406	13414	9
	46	TAG	3	3	13529	13537	9
	47	T	1	9	13993	14001	9
	48	A	1	8	14253	14260	8
	49	A	1	14	14727	14740	14
	50	T	1	13	15485	15497	13
	51	TA	2	4	15514	15521	8
	52	T	1	8	15526	15533	8
	53	ATT	3	3	15534	15542	9
	54	T	1	12	15653	15664	12
	55	AAC	3	3	15960	15968	9
	56	TTA	3	3	16770	16778	9
	57	T	1	8	17662	17669	8
	58	T	1	8	18331	18338	8
	59	T	1	11	19714	19724	11
	60	A	1	8	19857	19864	8
	61	T	1	10	22310	22319	10
	62	TTC	3	3	23326	23334	9
	63	A	1	8	23510	23517	8
	64	T	1	10	27375	27384	10
	65	AT	2	6	28278	28289	12
	66	T	1	8	29209	29216	8
	67	CTTG	4	3	29386	29397	12
	68	C	1	8	30767	30774	8
	69	T	1	10	31365	31374	10
	70	T	1	8	31604	31611	8
	71	A	1	9	31903	31911	9
	72	CT	2	4	31923	31930	8
	73	A	1	8	32707	32714	8
	74	A	1	12	32779	32790	12
	75	ATTA	4	3	33630	33641	12
	76	T	1	8	33946	33953	8
	77	A	1	8	34018	34025	8
	78	T	1	11	34671	34681	11
	79	T	1	9	34683	34691	9
	80	T	1	8	37867	37874	8
	81	GA	2	4	37978	37985	8
	82	T	1	8	38188	38195	8
	83	G	1	8	38619	38626	8
	84	AAT	3	3	38675	38683	9
	85	A	1	13	38728	38740	13
	86	AT	2	4	38822	38829	8

Species	Id	Motif	Type	Repeat	Start	End	Length
	87	TA	2	4	38834	38841	8
	88	TAA	3	3	39110	39118	9
	89	A	1	11	39362	39372	11
	90	TAA	3	3	39534	39542	9
	91	TCT	3	3	40111	40119	9
	92	ATG	3	3	43873	43881	9
	93	ATT	3	3	45168	45176	9
	94	AT	2	4	45280	45287	8
	95	A	1	8	45435	45442	8
	96	A	1	8	45460	45467	8
	97	T	1	8	45720	45727	8
	98	T	1	14	45804	45817	14
	99	TAAG	4	3	46066	46077	12
	100	T	1	10	47184	47193	10
	101	T	1	9	47221	47229	9
	102	A	1	12	47283	47294	12
	103	A	1	8	47323	47330	8
	104	A	1	8	47576	47583	8
	105	A	1	8	47589	47596	8
	106	A	1	9	47926	47934	9
	107	A	1	10	48083	48092	10
	108	A	1	9	48143	48151	9
	109	T	1	10	49786	49795	10
	110	TA	2	4	50063	50070	8
	111	AT	2	4	50075	50082	8
	112	A	1	8	50384	50391	8
	113	AT	2	4	50436	50443	8
	114	AT	2	4	50448	50455	8
	115	T	1	14	51285	51298	14
	116	T	1	14	53538	53551	14
	117	T	1	12	54145	54156	12
	118	T	1	8	54287	54294	8
	119	T	1	9	54545	54553	9
	120	A	1	8	55550	55557	8
	121	T	1	10	57460	57469	10
	122	ATA	3	3	57514	57522	9
	123	A	1	9	57566	57574	9
	124	T	1	9	57917	57925	9
	125	T	1	9	57936	57944	9
	126	TTG	3	3	58243	58251	9
	127	AT	2	4	58576	58583	8
	128	GA	2	4	58760	58767	8
	129	GCT	3	3	59654	59662	9
	130	A	1	8	59838	59845	8
	131	A	1	9	60146	60154	9
	132	AT	2	4	60191	60198	8

Species	Id	Motif	Type	Repeat	Start	End	Length
	133	AT	2	4	60643	60650	8
	134	AGT	3	3	60900	60908	9
	135	GGA	3	3	61262	61270	9
	136	A	1	8	62112	62119	8
	137	TA	2	4	62203	62210	8
	138	A	1	8	62365	62372	8
	139	AT	2	4	62499	62506	8
	140	T	1	9	62749	62757	9
	141	T	1	11	63298	63308	11
	142	GAA	3	3	63698	63706	9
	143	TCTT	4	3	63736	63747	12
	144	TC	2	5	64126	64135	10
	145	AT	2	4	65040	65047	8
	146	C	1	8	65326	65333	8
	147	A	1	8	65400	65407	8
	148	CTT	3	3	66320	66328	9
	149	AT	2	4	66645	66652	8
	150	T	1	10	66798	66807	10
	151	TTA	3	3	66928	66936	9
	152	TTA	3	3	67173	67181	9
	153	T	1	9	68120	68128	9
	154	A	1	9	68193	68201	9
	155	A	1	8	68617	68624	8
	156	TTA	3	3	68681	68689	9
	157	A	1	8	69567	69574	8
	158	T	1	9	69888	69896	9
	159	ATA	3	3	69912	69920	9
	160	AT	2	4	70117	70124	8
	161	T	1	8	70335	70342	8
	162	T	1	10	70427	70436	10
	163	T	1	8	70689	70696	8
	164	A	1	8	71061	71068	8
	165	AAC	3	3	71322	71330	9
	166	TAAA	4	3	71448	71459	12
	167	A	1	9	71465	71473	9
	168	A	1	8	71564	71571	8
	169	T	1	12	72723	72734	12
	170	T	1	8	72827	72834	8
	171	T	1	10	73137	73146	10
	172	T	1	11	73691	73701	11
	173	A	1	9	73875	73883	9
	174	T	1	10	73957	73966	10
	175	A	1	9	74357	74365	9
	176	T	1	10	74396	74405	10
	177	T	1	9	74457	74465	9
	178	A	1	9	74825	74833	9

Species	Id	Motif	Type	Repeat	Start	End	Length
	179	A	1	8	75332	75339	8
	180	A	1	8	75492	75499	8
	181	T	1	8	75564	75571	8
	182	T	1	8	76359	76366	8
	183	TTG	3	3	76374	76382	9
	184	TCT	3	3	77007	77015	9
	185	TTTC	4	3	78001	78012	12
	186	T	1	8	78264	78271	8
	187	TTC	3	3	81368	81376	9
	188	ATA	3	3	82071	82079	9
	189	T	1	10	83590	83599	10
	190	A	1	11	84133	84143	11
	191	T	1	8	84178	84185	8
	192	TA	2	4	84803	84810	8
	193	T	1	8	85490	85497	8
	194	T	1	8	85569	85576	8
	195	T	1	8	85677	85684	8
	196	T	1	9	85715	85723	9
	197	T	1	9	85730	85738	9
	198	T	1	10	86479	86488	10
	199	TGC	3	3	86711	86719	9
	200	AT	2	4	86848	86855	8
	201	T	1	9	87244	87252	9
	202	T	1	8	87282	87289	8
	203	CTT	3	3	87378	87386	9
	204	TA	2	4	87870	87877	8
	205	GA	2	4	89487	89494	8
	206	GA	2	4	89499	89506	8
	207	GA	2	4	90486	90493	8
	208	ATT	3	3	92253	92261	9
	209	A	1	8	92669	92676	8
	210	GA	2	4	92689	92696	8
	211	TCT	3	3	93372	93380	9
	212	CTT	3	3	93478	93486	9
	213	GGT	3	3	93742	93750	9
	214	GAA	3	3	95223	95231	9
	215	TA	2	4	96060	96067	8
	216	TA	2	4	97503	97510	8
	217	AG	2	4	98263	98270	8
	218	AGA	3	3	98558	98566	9
	219	T	1	8	98904	98911	8
	220	AGA	3	3	99998	100006	9
	221	T	1	8	102211	102218	8
	222	G	1	9	103096	103104	9
	223	AAG	3	3	103278	103286	9
	224	TTC	3	4	106536	106547	12

Species	Id	Motif	Type	Repeat	Start	End	Length
	225	CTG	3	3	107990	107998	9
	226	CT	2	4	109551	109558	8
	227	AT	2	4	111263	111270	8
	228	A	1	12	111279	111290	12
	229	T	1	9	111431	111439	9
	230	CAA	3	3	113051	113059	9
	231	T	1	10	113620	113629	10
	232	A	1	8	113653	113660	8
	233	A	1	8	114385	114392	8
	234	ATT	3	3	114404	114412	9
	235	TAA	3	3	115337	115345	9
	236	ATAG	4	3	115731	115742	12
	237	CTT	3	3	116037	116045	9
	238	A	1	14	116350	116363	14
	239	T	1	8	116482	116489	8
	240	A	1	9	116812	116820	9
	241	A	1	10	116822	116831	10
	242	A	1	9	116932	116940	9
	243	T	1	11	117199	117209	11
	244	A	1	9	117430	117438	9
	245	AAT	3	3	117477	117485	9
	246	T	1	8	117771	117778	8
	247	A	1	8	118220	118227	8
	248	T	1	8	118306	118313	8
	249	T	1	8	118858	118865	8
	250	T	1	9	118883	118891	9
	251	AAT	3	3	118916	118924	9
	252	AATA	4	3	119164	119175	12
	253	T	1	8	119555	119562	8
	254	A	1	8	120489	120496	8
	255	A	1	8	120561	120568	8
	256	A	1	8	122508	122515	8
	257	T	1	8	122598	122605	8
	258	TTA	3	3	122609	122617	9
	259	AT	2	4	123907	123914	8
	260	ATA	3	3	123980	123988	9
	261	A	1	10	124126	124135	10
	262	T	1	10	124200	124209	10
	263	T	1	10	124604	124613	10
	264	A	1	8	126606	126613	8
	265	T	1	8	126933	126940	8
	266	T	1	9	127066	127074	9
	267	TAA	3	3	127162	127170	9
	268	AT	2	4	127321	127328	8
	269	T	1	8	127743	127750	8
	270	T	1	8	128284	128291	8

Species	Id	Motif	Type	Repeat	Start	End	Length
	271	T	1	9	128515	128523	9
	272	ATC	3	3	128761	128769	9
	273	TAAT	4	3	129014	129025	12
	274	T	1	9	129461	129469	9
	275	T	1	8	129519	129526	8
	276	A	1	8	129535	129542	8
	277	T	1	9	129831	129839	9
	278	TTA	3	3	130002	130010	9
	279	A	1	9	130067	130075	9
	280	T	1	8	130134	130141	8
	281	T	1	9	130654	130662	9
	282	TC	2	4	131063	131070	8
	283	T	1	9	131146	131154	9
	284	T	1	8	131263	131270	8
	285	A	1	9	131421	131429	9
	286	A	1	8	131766	131773	8
	287	TTG	3	3	132461	132469	9
	288	A	1	9	134081	134089	9
	289	T	1	12	134230	134241	12
	290	AT	2	4	134250	134257	8
	291	AG	2	4	135962	135969	8
	292	CAG	3	3	137522	137530	9
	293	AGA	3	4	138972	138983	12
	294	CTT	3	3	142234	142242	9
	295	C	1	9	142416	142424	9
	296	A	1	8	143302	143309	8
	297	TTC	3	3	145513	145521	9
	298	A	1	8	146609	146616	8
	299	TCT	3	3	146954	146962	9
	300	CT	2	4	147250	147257	8
	301	AT	2	4	148009	148016	8
	302	TA	2	4	149453	149460	8
	303	TTC	3	3	150289	150297	9
	304	ACC	3	3	151770	151778	9
	305	AAG	3	3	152034	152042	9
	306	AGA	3	3	152140	152148	9
	307	TC	2	4	152824	152831	8
	308	T	1	8	152844	152851	8
	309	AAT	3	3	153259	153267	9
	310	TC	2	4	155027	155034	8
	311	TC	2	4	156014	156021	8
	312	TC	2	4	156026	156033	8
	313	AT	2	4	157642	157649	8
	314	GAA	3	3	158133	158141	9
	315	A	1	8	158231	158238	8
Eugenia nitida	1	A	1	9	17	25	9

Species	Id	Motif	Type	Repeat	Start	End	Length
	2	T	1	10	150	159	10
	3	A	1	9	314	322	9
	4	CAG	3	4	1187	1198	12
	5	A	1	8	1995	2002	8
	6	T	1	8	2171	2178	8
	7	TCC	3	3	2347	2355	9
	8	T	1	9	2482	2490	9
	9	CA	2	4	3101	3108	8
	10	A	1	10	3773	3782	10
	11	T	1	10	4294	4303	10
	12	A	1	8	4532	4539	8
	13	A	1	9	4705	4713	9
	14	A	1	8	4766	4773	8
	15	TA	2	4	4814	4821	8
	16	T	1	9	4826	4834	9
	17	AGAT	4	3	4874	4885	12
	18	A	1	8	5665	5672	8
	19	A	1	9	6547	6555	9
	20	A	1	8	6705	6712	8
	21	A	1	8	6774	6781	8
	22	TA	2	4	7519	7526	8
	23	T	1	10	7872	7881	10
	24	A	1	10	7950	7959	10
	25	A	1	9	8187	8195	9
	26	T	1	9	8348	8356	9
	27	T	1	10	8478	8487	10
	28	A	1	13	8489	8501	13
	29	T	1	9	9048	9056	9
	30	A	1	9	9297	9305	9
	31	T	1	10	9519	9528	10
	32	T	1	9	9824	9832	9
	33	AT	2	4	10479	10486	8
	34	A	1	10	10657	10666	10
	35	A	1	9	10674	10682	9
	36	T	1	10	10901	10910	10
	37	A	1	10	10973	10982	10
	38	ATTA	4	3	10993	11004	12
	39	ATTT	4	3	11041	11052	12
	40	A	1	9	12607	12615	9
	41	T	1	10	13166	13175	10
	42	A	1	11	13393	13403	11
	43	TAG	3	3	13518	13526	9
	44	T	1	9	13982	13990	9
	45	A	1	8	14232	14239	8
	46	A	1	9	14414	14422	9
	47	A	1	10	14707	14716	10

Species	Id	Motif	Type	Repeat	Start	End	Length
	48	T	1	9	15456	15464	9
	49	T	1	10	15466	15475	10
	50	TA	2	4	15492	15499	8
	51	T	1	8	15504	15511	8
	52	ATT	3	3	15512	15520	9
	53	A	1	8	15530	15537	8
	54	T	1	8	15631	15638	8
	55	AAC	3	3	15934	15942	9
	56	TTA	3	3	16744	16752	9
	57	A	1	12	17490	17501	12
	58	T	1	8	17659	17666	8
	59	T	1	8	18328	18335	8
	60	T	1	11	19711	19721	11
	61	A	1	8	19854	19861	8
	62	T	1	10	22307	22316	10
	63	TTC	3	3	23323	23331	9
	64	A	1	8	23507	23514	8
	65	C	1	8	26675	26682	8
	66	T	1	10	27373	27382	10
	67	AT	2	4	28276	28283	8
	68	A	1	9	28284	28292	9
	69	T	1	8	29205	29212	8
	70	CTTG	4	3	29382	29393	12
	71	C	1	10	30765	30774	10
	72	T	1	8	31365	31372	8
	73	T	1	8	31602	31609	8
	74	A	1	9	31901	31909	9
	75	CT	2	4	31921	31928	8
	76	A	1	12	32311	32322	12
	77	A	1	8	32705	32712	8
	78	A	1	9	32777	32785	9
	79	AATGG	5	3	33256	33270	15
	80	T	1	8	33947	33954	8
	81	T	1	10	34655	34664	10
	82	T	1	10	34671	34680	10
	83	T	1	8	37545	37552	8
	84	GA	2	4	37656	37663	8
	85	A	1	8	38392	38399	8
	86	G	1	8	38404	38411	8
	87	A	1	8	38643	38650	8
	88	C	1	9	38792	38800	9
	89	TAA	3	3	38814	38822	9
	90	TCT	3	3	39392	39400	9
	91	ATG	3	3	43154	43162	9
	92	AT	2	4	44533	44540	8
	93	A	1	8	44688	44695	8

Species	Id	Motif	Type	Repeat	Start	End	Length
	94	A	1	8	44713	44720	8
	95	T	1	9	44973	44981	9
	96	T	1	10	45057	45066	10
	97	TAAG	4	3	45315	45326	12
	98	T	1	12	46440	46451	12
	99	T	1	10	46479	46488	10
	100	A	1	10	46542	46551	10
	101	A	1	8	46580	46587	8
	102	A	1	11	46833	46843	11
	103	A	1	8	46849	46856	8
	104	A	1	9	47177	47185	9
	105	A	1	11	47334	47344	11
	106	A	1	10	47394	47403	10
	107	T	1	10	49038	49047	10
	108	TA	2	4	49346	49353	8
	109	AT	2	4	49358	49365	8
	110	A	1	9	49689	49697	9
	111	AT	2	4	49777	49784	8
	112	AT	2	4	49789	49796	8
	113	T	1	10	50625	50634	10
	114	GT	2	4	51313	51320	8
	115	T	1	9	51453	51461	9
	116	T	1	14	52878	52891	14
	117	T	1	11	53485	53495	11
	118	T	1	10	53626	53635	10
	119	T	1	11	53886	53896	11
	120	A	1	9	54893	54901	9
	121	T	1	10	56804	56813	10
	122	ATA	3	3	56858	56866	9
	123	A	1	10	56910	56919	10
	124	T	1	8	57262	57269	8
	125	T	1	9	57280	57288	9
	126	TTG	3	3	57587	57595	9
	127	AT	2	4	57920	57927	8
	128	GA	2	4	58104	58111	8
	129	GCT	3	3	58998	59006	9
	130	A	1	8	59207	59214	8
	131	A	1	10	59515	59524	10
	132	AT	2	4	59561	59568	8
	133	AT	2	4	60013	60020	8
	134	AGT	3	3	60270	60278	9
	135	GGA	3	3	60632	60640	9
	136	T	1	10	61444	61453	10
	137	A	1	8	61472	61479	8
	138	A	1	8	61608	61615	8
	139	AT	2	4	61742	61749	8

Species	Id	Motif	Type	Repeat	Start	End	Length
	140	T	1	8	61992	61999	8
	141	T	1	11	62540	62550	11
	142	TCTT	4	3	62978	62989	12
	143	TC	2	5	63361	63370	10
	144	AT	2	4	64275	64282	8
	145	C	1	8	64561	64568	8
	146	A	1	8	64635	64642	8
	147	CTT	3	3	65555	65563	9
	148	AT	2	4	65875	65882	8
	149	T	1	10	66028	66037	10
	150	TTA	3	3	66158	66166	9
	151	TTA	3	3	66403	66411	9
	152	T	1	10	67350	67359	10
	153	A	1	9	67424	67432	9
	154	A	1	8	67442	67449	8
	155	A	1	8	67843	67850	8
	156	TTA	3	3	67907	67915	9
	157	AT	2	4	68459	68466	8
	158	T	1	8	68631	68638	8
	159	A	1	10	68827	68836	10
	160	T	1	8	69150	69157	8
	161	ATA	3	3	69173	69181	9
	162	AT	2	4	69379	69386	8
	163	T	1	8	69597	69604	8
	164	T	1	10	69689	69698	10
	165	T	1	9	69952	69960	9
	166	TA	2	4	70275	70282	8
	167	A	1	8	70326	70333	8
	168	AAC	3	3	70587	70595	9
	169	ATAA	4	3	70712	70723	12
	170	A	1	8	70730	70737	8
	171	A	1	8	70828	70835	8
	172	T	1	10	71998	72007	10
	173	T	1	9	72100	72108	9
	174	T	1	9	72411	72419	9
	175	T	1	11	72956	72966	11
	176	A	1	10	73139	73148	10
	177	T	1	11	73221	73231	11
	178	A	1	10	73622	73631	10
	179	T	1	9	73662	73670	9
	180	T	1	9	73722	73730	9
	181	A	1	10	74090	74099	10
	182	A	1	8	74753	74760	8
	183	T	1	8	74828	74835	8
	184	T	1	8	75623	75630	8
	185	TTG	3	3	75638	75646	9

**Species**	**Id**	**Motif**	**Type**	**Repeat**	**Start**	**End**	**Length**
	186	TCT	3	3	76271	76279	9
	187	TTTC	4	4	77261	77276	16
	188	TTC	3	3	80622	80630	9
	189	ATA	3	3	81325	81333	9
	190	T	1	10	82844	82853	10
	191	A	1	18	83387	83404	18
	192	T	1	8	83439	83446	8
	193	TTTC	4	3	84633	84644	12
	194	T	1	11	84946	84956	11
	195	T	1	8	84983	84990	8
	196	T	1	10	84997	85006	10
	197	T	1	10	85747	85756	10
	198	TGC	3	3	85979	85987	9
	199	AT	2	4	86116	86123	8
	200	T	1	9	86512	86520	9
	201	T	1	8	86550	86557	8
	202	CTT	3	3	86646	86654	9
	203	TA	2	4	87138	87145	8
	204	GA	2	4	88755	88762	8
	205	GA	2	4	88767	88774	8
	206	GA	2	4	89754	89761	8
	207	ATT	3	3	91542	91550	9
	208	A	1	9	91957	91965	9
	209	GA	2	4	91978	91985	8
	210	TCT	3	3	92661	92669	9
	211	CTT	3	3	92767	92775	9
	212	GGT	3	3	93031	93039	9
	213	GAA	3	3	94512	94520	9
	214	TA	2	4	95349	95356	8
	215	TA	2	4	96792	96799	8
	216	AG	2	4	97552	97559	8
	217	AGA	3	3	97847	97855	9
	218	T	1	9	98193	98201	9
	219	AGA	3	3	99288	99296	9
	220	T	1	8	101501	101508	8
	221	AAG	3	3	102566	102574	9
	222	T	1	8	105497	105504	8
	223	TTC	3	4	105825	105836	12
	224	CTG	3	3	107280	107288	9
	225	CT	2	4	108845	108852	8
	226	AT	2	4	110557	110564	8
	227	A	1	10	110573	110582	10
	228	T	1	9	110724	110732	9
	229	CAA	3	3	112344	112352	9
	230	TTA	3	5	112965	112979	15
	231	T	1	11	113017	113027	11

Species	Id	Motif	Type	Repeat	Start	End	Length
	232	T	1	10	113029	113038	10
	233	A	1	8	113781	113788	8
	234	ATT	3	3	113800	113808	9
	235	TAA	3	3	114733	114741	9
	236	ATAG	4	3	115127	115138	12
	237	ATT	3	3	115609	115617	9
	238	A	1	14	115746	115759	14
	239	A	1	9	116206	116214	9
	240	A	1	8	116216	116223	8
	241	T	1	10	116239	116248	10
	242	A	1	10	116327	116336	10
	243	T	1	9	116594	116602	9
	244	A	1	11	116823	116833	11
	245	AAT	3	3	116874	116882	9
	246	T	1	8	117168	117175	8
	247	T	1	8	117241	117248	8
	248	A	1	8	117617	117624	8
	249	T	1	8	117703	117710	8
	250	A	1	9	118071	118079	9
	251	T	1	8	118257	118264	8
	252	T	1	9	118282	118290	9
	253	AAT	3	3	118315	118323	9
	254	AATA	4	3	118563	118574	12
	255	T	1	8	118954	118961	8
	256	A	1	8	119888	119895	8
	257	A	1	8	119960	119967	8
	258	A	1	8	121908	121915	8
	259	T	1	9	121998	122006	9
	260	TTA	3	3	122010	122018	9
	261	AT	2	5	123314	123323	10
	262	ATA	3	3	123388	123396	9
	263	A	1	10	123534	123543	10
	264	T	1	9	123608	123616	9
	265	T	1	12	124011	124022	12
	266	A	1	8	124715	124722	8
	267	A	1	8	126014	126021	8
	268	T	1	9	126341	126349	9
	269	TAA	3	3	126569	126577	9
	270	AT	2	4	126728	126735	8
	271	T	1	8	127150	127157	8
	272	T	1	8	127691	127698	8
	273	T	1	9	127922	127930	9
	274	ATC	3	3	128168	128176	9
	275	TAAT	4	3	128421	128432	12
	276	T	1	8	128926	128933	8
	277	A	1	8	128942	128949	8

Species	Id	Motif	Type	Repeat	Start	End	Length
	278	T	1	9	129238	129246	9
	279	TTA	3	3	129409	129417	9
	280	A	1	11	129473	129483	11
	281	T	1	8	129541	129548	8
	282	T	1	9	130061	130069	9
	283	TC	2	4	130470	130477	8
	284	T	1	10	130553	130562	10
	285	T	1	8	130670	130677	8
	286	A	1	9	130828	130836	9
	287	A	1	8	131173	131180	8
	288	TTG	3	3	131868	131876	9
	289	A	1	9	133488	133496	9
	290	T	1	10	133638	133647	10
	291	AT	2	4	133656	133663	8
	292	AG	2	4	135368	135375	8
	293	CAG	3	3	136932	136940	9
	294	AGA	3	4	138383	138394	12
	295	A	1	8	138716	138723	8
	296	CTT	3	3	141646	141654	9
	297	A	1	8	142712	142719	8
	298	TTC	3	3	144923	144931	9
	299	A	1	9	146019	146027	9
	300	TCT	3	3	146365	146373	9
	301	CT	2	4	146661	146668	8
	302	AT	2	4	147420	147427	8
	303	TA	2	4	148864	148871	8
	304	TTC	3	3	149700	149708	9
	305	ACC	3	3	151181	151189	9
	306	AAG	3	3	151445	151453	9
	307	AGA	3	3	151551	151559	9
	308	TC	2	4	152235	152242	8
	309	T	1	9	152255	152263	9
	310	AAT	3	3	152670	152678	9
	311	TC	2	4	154459	154466	8
	312	TC	2	4	155446	155453	8
	313	TC	2	4	155458	155465	8
	314	AT	2	4	157074	157081	8
	315	GAA	3	3	157565	157573	9
	316	A	1	8	157663	157670	8
Eugenia pyriformis	1	A	1	9	17	25	9
	2	T	1	9	133	141	9
	3	A	1	8	291	298	8
	4	CAG	3	4	1163	1174	12
	5	A	1	8	1971	1978	8
	6	T	1	9	2147	2155	9
	7	TCC	3	3	2321	2329	9

Species	Id	Motif	Type	Repeat	Start	End	Length
	8	T	1	9	2456	2464	9
	9	CA	2	4	3075	3082	8
	10	A	1	10	3747	3756	10
	11	A	1	10	4502	4511	10
	12	A	1	9	4677	4685	9
	13	A	1	11	4737	4747	11
	14	TA	2	4	4788	4795	8
	15	T	1	8	4800	4807	8
	16	AGAT	4	3	4851	4862	12
	17	A	1	9	4934	4942	9
	18	A	1	8	5642	5649	8
	19	T	1	9	6357	6365	9
	20	A	1	8	6664	6671	8
	21	A	1	8	6733	6740	8
	22	TA	2	4	7478	7485	8
	23	T	1	11	7831	7841	11
	24	A	1	8	7910	7917	8
	25	A	1	9	8145	8153	9
	26	T	1	9	8306	8314	9
	27	T	1	10	8436	8445	10
	28	A	1	11	8447	8457	11
	29	A	1	12	8671	8682	12
	30	A	1	9	8699	8707	9
	31	A	1	9	8870	8878	9
	32	A	1	11	9005	9015	11
	33	T	1	9	9052	9060	9
	34	TAT	3	3	9253	9261	9
	35	ATT	3	3	9517	9525	9
	36	T	1	10	9838	9847	10
	37	AT	2	4	10477	10484	8
	38	A	1	12	10655	10666	12
	39	A	1	10	10674	10683	10
	40	T	1	9	10907	10915	9
	41	A	1	10	10982	10991	10
	42	ATTA	4	3	11002	11013	12
	43	ATTT	4	3	11050	11061	12
	44	A	1	9	12616	12624	9
	45	T	1	9	13175	13183	9
	46	A	1	10	13401	13410	10
	47	TAG	3	3	13525	13533	9
	48	T	1	9	13989	13997	9
	49	A	1	8	14250	14257	8
	50	A	1	9	14432	14440	9
	51	A	1	10	14726	14735	10
	52	T	1	10	15468	15477	10
	53	T	1	10	15479	15488	10

Species	Id	Motif	Type	Repeat	Start	End	Length
	54	AT	2	6	15502	15513	12
	55	TTA	3	3	15522	15530	9
	56	TTA	3	3	15542	15550	9
	57	T	1	11	15663	15673	11
	58	AAC	3	3	15969	15977	9
	59	TTA	3	3	16779	16787	9
	60	T	1	9	17519	17527	9
	61	T	1	8	17687	17694	8
	62	T	1	8	18356	18363	8
	63	T	1	11	19739	19749	11
	64	A	1	8	19882	19889	8
	65	T	1	10	22335	22344	10
	66	TTC	3	3	23351	23359	9
	67	A	1	8	23535	23542	8
	68	T	1	10	27400	27409	10
	69	AT	2	4	28303	28310	8
	70	A	1	8	28311	28318	8
	71	TTA	3	3	28329	28337	9
	72	C	1	9	29180	29188	9
	73	T	1	8	29246	29253	8
	74	CTTG	4	3	29423	29434	12
	75	T	1	10	30576	30585	10
	76	T	1	8	31406	31413	8
	77	T	1	8	31643	31650	8
	78	A	1	9	31942	31950	9
	79	CT	2	4	31962	31969	8
	80	A	1	15	32345	32359	15
	81	A	1	9	32742	32750	9
	82	A	1	8	32815	32822	8
	83	ATTA	4	3	33666	33677	12
	84	T	1	10	34672	34681	10
	85	T	1	8	37874	37881	8
	86	GA	2	4	37985	37992	8
	87	A	1	9	38710	38718	9
	88	G	1	8	38723	38730	8
	89	A	1	11	38832	38842	11
	90	AT	2	4	38924	38931	8
	91	TA	2	5	38936	38945	10
	92	A	1	8	39465	39472	8
	93	C	1	8	39614	39621	8
	94	TAA	3	3	39635	39643	9
	95	TCT	3	3	40219	40227	9
	96	ATG	3	3	43972	43980	9
	97	AGT	3	3	44725	44733	9
	98	T	1	8	45124	45131	8
	99	A	1	8	45434	45441	8

Species	Id	Motif	Type	Repeat	Start	End	Length
	100	A	1	8	45459	45466	8
	101	T	1	10	45801	45810	10
	102	TAAG	4	3	46059	46070	12
	103	T	1	11	47177	47187	11
	104	T	1	11	47215	47225	11
	105	A	1	8	47315	47322	8
	106	A	1	11	47568	47578	11
	107	A	1	8	47584	47591	8
	108	A	1	8	47912	47919	8
	109	A	1	9	48068	48076	9
	110	A	1	10	48127	48136	10
	111	T	1	9	49779	49787	9
	112	TA	2	4	50073	50080	8
	113	AT	2	4	50085	50092	8
	114	A	1	8	50412	50419	8
	115	AT	2	4	50487	50494	8
	116	T	1	12	51329	51340	12
	117	T	1	11	53580	53590	11
	118	T	1	8	54170	54177	8
	119	T	1	9	54308	54316	9
	120	T	1	14	54567	54580	14
	121	A	1	9	55578	55586	9
	122	T	1	10	57489	57498	10
	123	ATA	3	3	57543	57551	9
	124	A	1	9	57594	57602	9
	125	T	1	8	57945	57952	8
	126	T	1	10	57963	57972	10
	127	TTG	3	3	58271	58279	9
	128	AT	2	4	58604	58611	8
	129	GA	2	4	58788	58795	8
	130	GCT	3	3	59682	59690	9
	131	A	1	9	59866	59874	9
	132	A	1	9	60174	60182	9
	133	AT	2	4	60219	60226	8
	134	AT	2	4	60671	60678	8
	135	AGT	3	3	60928	60936	9
	136	GGA	3	3	61290	61298	9
	137	TTTAT	5	3	61925	61939	15
	138	A	1	8	62140	62147	8
	139	TA	2	4	62231	62238	8
	140	A	1	8	62393	62400	8
	141	AT	2	4	62527	62534	8
	142	T	1	9	62777	62785	9
	143	T	1	11	63327	63337	11
	144	GAA	3	3	63727	63735	9
	145	TCTT	4	3	63765	63776	12

Species	Id	Motif	Type	Repeat	Start	End	Length
	146	TC	2	5	64148	64157	10
	147	AT	2	4	65056	65063	8
	148	C	1	8	65342	65349	8
	149	A	1	8	65416	65423	8
	150	CTT	3	3	66318	66326	9
	151	AT	2	4	66658	66665	8
	152	T	1	11	66811	66821	11
	153	TTA	3	3	66942	66950	9
	154	TTA	3	3	67187	67195	9
	155	T	1	9	68135	68143	9
	156	A	1	10	68202	68211	10
	157	A	1	8	68221	68228	8
	158	A	1	8	68633	68640	8
	159	T	1	8	69393	69400	8
	160	A	1	9	69596	69604	9
	161	T	1	8	69920	69927	8
	162	ATA	3	3	69943	69951	9
	163	AT	2	4	70149	70156	8
	164	T	1	8	70366	70373	8
	165	T	1	15	70458	70472	15
	166	T	1	9	70681	70689	9
	167	TA	2	4	71004	71011	8
	168	AAC	3	3	71315	71323	9
	169	ATAA	4	3	71440	71451	12
	170	A	1	8	71458	71465	8
	171	A	1	8	72305	72312	8
	172	T	1	13	72714	72726	13
	173	T	1	8	72819	72826	8
	174	T	1	13	73682	73694	13
	175	A	1	9	73868	73876	9
	176	T	1	9	73950	73958	9
	177	A	1	9	74349	74357	9
	178	T	1	13	74388	74400	13
	179	T	1	9	74452	74460	9
	180	A	1	11	74820	74830	11
	181	A	1	8	75483	75490	8
	182	T	1	8	75555	75562	8
	183	T	1	8	76350	76357	8
	184	TTG	3	3	76365	76373	9
	185	TCT	3	3	76998	77006	9
	186	TTTC	4	3	77989	78000	12
	187	T	1	8	78251	78258	8
	188	T	1	9	80277	80285	9
	189	TTC	3	3	81352	81360	9
	190	T	1	8	81849	81856	8
	191	ATA	3	3	82055	82063	9

Species	Id	Motif	Type	Repeat	Start	End	Length
	192	T	1	10	82979	82988	10
	193	T	1	13	83577	83589	13
	194	A	1	10	84110	84119	10
	195	T	1	9	84154	84162	9
	196	TA	2	4	84776	84783	8
	197	TTTC	4	3	85349	85360	12
	198	T	1	8	85476	85483	8
	199	T	1	10	85662	85671	10
	200	T	1	8	85702	85709	8
	201	T	1	9	85716	85724	9
	202	T	1	10	86465	86474	10
	203	TGC	3	3	86697	86705	9
	204	AT	2	4	86834	86841	8
	205	T	1	9	87230	87238	9
	206	T	1	8	87268	87275	8
	207	CTT	3	3	87364	87372	9
	208	TA	2	4	87856	87863	8
	209	GA	2	4	89473	89480	8
	210	GA	2	4	89485	89492	8
	211	GA	2	4	90472	90479	8
	212	ATT	3	3	92239	92247	9
	213	A	1	9	92654	92662	9
	214	GA	2	4	92675	92682	8
	215	TCT	3	3	93358	93366	9
	216	CTT	3	3	93464	93472	9
	217	GGT	3	3	93728	93736	9
	218	GAA	3	3	95209	95217	9
	219	TA	2	4	96046	96053	8
	220	TA	2	4	97489	97496	8
	221	AG	2	4	98249	98256	8
	222	AGA	3	3	98544	98552	9
	223	T	1	8	98890	98897	8
	224	AGA	3	3	99984	99992	9
	225	T	1	8	102202	102209	8
	226	AAG	3	3	103267	103275	9
	227	T	1	8	106198	106205	8
	228	TTC	3	4	106526	106537	12
	229	CTG	3	3	107979	107987	9
	230	CT	2	4	109540	109547	8
	231	AT	2	4	111252	111259	8
	232	A	1	9	111268	111276	9
	233	T	1	9	111418	111426	9
	234	CAA	3	3	113038	113046	9
	235	ATT	3	3	113656	113664	9
	236	T	1	10	113703	113712	10
	237	T	1	8	113714	113721	8

Species	Id	Motif	Type	Repeat	Start	End	Length
	238	A	1	8	113741	113748	8
	239	ATT	3	3	114492	114500	9
	240	TAA	3	3	115425	115433	9
	241	ATAG	4	3	115819	115830	12
	242	ATT	3	3	116301	116309	9
	243	A	1	8	116429	116436	8
	244	T	1	8	116447	116454	8
	245	A	1	12	117138	117149	12
	246	T	1	8	117165	117172	8
	247	A	1	11	117251	117261	11
	248	A	1	10	117747	117756	10
	249	AAT	3	3	117795	117803	9
	250	T	1	8	118096	118103	8
	251	A	1	8	118545	118552	8
	252	T	1	8	118631	118638	8
	253	A	1	9	118999	119007	9
	254	A	1	8	119055	119062	8
	255	T	1	8	119185	119192	8
	256	T	1	8	119210	119217	8
	257	AAT	3	3	119242	119250	9
	258	AATA	4	3	119483	119494	12
	259	T	1	8	119874	119881	8
	260	A	1	8	120808	120815	8
	261	A	1	8	120880	120887	8
	262	A	1	8	122829	122836	8
	263	TTA	3	3	122931	122939	9
	264	AT	2	4	124229	124236	8
	265	ATA	3	3	124302	124310	9
	266	A	1	10	124448	124457	10
	267	T	1	10	124522	124531	10
	268	T	1	11	124926	124936	11
	269	A	1	11	126928	126938	11
	270	T	1	8	127258	127265	8
	271	TAA	3	3	127485	127493	9
	272	AT	2	4	127644	127651	8
	273	T	1	8	128066	128073	8
	274	T	1	8	128607	128614	8
	275	ATC	3	3	129084	129092	9
	276	TAAT	4	3	129337	129348	12
	277	T	1	8	129784	129791	8
	278	T	1	12	129795	129806	12
	279	T	1	8	129842	129849	8
	280	A	1	8	129858	129865	8
	281	T	1	9	130154	130162	9
	282	A	1	11	130383	130393	11
	283	T	1	8	130451	130458	8

Species	Id	Motif	Type	Repeat	Start	End	Length
	284	T	1	9	130971	130979	9
	285	TC	2	4	131380	131387	8
	286	T	1	9	131579	131587	9
	287	A	1	10	131738	131747	10
	288	A	1	8	132083	132090	8
	289	TTG	3	3	132778	132786	9
	290	A	1	9	134398	134406	9
	291	T	1	9	134548	134556	9
	292	AT	2	4	134565	134572	8
	293	AG	2	4	136277	136284	8
	294	CAG	3	3	137837	137845	9
	295	AGA	3	4	139286	139297	12
	296	A	1	8	139619	139626	8
	297	CTT	3	3	142549	142557	9
	298	A	1	8	143615	143622	8
	299	TTC	3	3	145831	145839	9
	300	A	1	8	146927	146934	8
	301	TCT	3	3	147272	147280	9
	302	CT	2	4	147568	147575	8
	303	AT	2	4	148327	148334	8
	304	TA	2	4	149771	149778	8
	305	TTC	3	3	150607	150615	9
	306	ACC	3	3	152088	152096	9
	307	AAG	3	3	152352	152360	9
	308	AGA	3	3	152458	152466	9
	309	TC	2	4	153142	153149	8
	310	T	1	9	153162	153170	9
	311	AAT	3	3	153577	153585	9
	312	TC	2	4	155345	155352	8
	313	TC	2	4	156332	156339	8
	314	TC	2	4	156344	156351	8
	315	AT	2	4	157960	157967	8
	316	GAA	3	3	158451	158459	9
	317	A	1	8	158549	158556	8
Myrcianthes pungens	1	A	1	9	17	25	9
	2	A	1	9	304	312	9
	3	CAG	3	4	1177	1188	12
	4	A	1	8	1985	1992	8
	5	T	1	8	2154	2161	8
	6	TCC	3	3	2330	2338	9
	7	T	1	9	2465	2473	9
	8	CA	2	4	3084	3091	8
	9	A	1	11	3756	3766	11
	10	A	1	10	4512	4521	10
	11	A	1	9	4681	4689	9
	12	T	1	9	4731	4739	9

Species	Id	Motif	Type	Repeat	Start	End	Length
	13	A	1	8	4744	4751	8
	14	TA	2	4	4792	4799	8
	15	T	1	10	4804	4813	10
	16	AGAT	4	3	4858	4869	12
	17	A	1	8	4944	4951	8
	18	C	1	8	5321	5328	8
	19	A	1	8	5651	5658	8
	20	GA	2	4	5981	5988	8
	21	A	1	8	6531	6538	8
	22	A	1	9	6688	6696	9
	23	A	1	8	6758	6765	8
	24	TA	2	4	7554	7561	8
	25	T	1	11	7907	7917	11
	26	A	1	9	7986	7994	9
	27	A	1	11	8222	8232	11
	28	T	1	10	8390	8399	10
	29	T	1	13	8521	8533	13
	30	A	1	11	8535	8545	11
	31	A	1	10	8763	8772	10
	32	A	1	9	8794	8802	9
	33	A	1	10	8809	8818	10
	34	A	1	8	8981	8988	8
	35	A	1	10	9100	9109	10
	36	T	1	12	9155	9166	12
	37	TAT	3	3	9359	9367	9
	38	A	1	9	9395	9403	9
	39	T	1	10	9626	9635	10
	40	T	1	8	9944	9951	8
	41	AT	2	4	10593	10600	8
	42	A	1	10	10792	10801	10
	43	T	1	9	11020	11028	9
	44	A	1	10	11091	11100	10
	45	ATTA	4	3	11111	11122	12
	46	ATTT	4	3	11159	11170	12
	47	A	1	9	12725	12733	9
	48	T	1	9	13284	13292	9
	49	A	1	9	13510	13518	9
	50	TAG	3	3	13633	13641	9
	51	T	1	10	14098	14107	10
	52	ATT	3	3	14192	14200	9
	53	A	1	8	14359	14366	8
	54	A	1	8	14531	14538	8
	55	A	1	12	14823	14834	12
	56	T	1	14	15545	15558	14
	57	TA	2	4	15571	15578	8
	58	T	1	8	15583	15590	8

Species	Id	Motif	Type	Repeat	Start	End	Length
	59	T	1	12	15710	15721	12
	60	A	1	9	15857	15865	9
	61	AAC	3	3	16020	16028	9
	62	TTA	3	3	16830	16838	9
	63	T	1	12	17570	17581	12
	64	T	1	9	17743	17751	9
	65	T	1	8	18413	18420	8
	66	T	1	11	19796	19806	11
	67	A	1	8	19939	19946	8
	68	T	1	10	22392	22401	10
	69	TTC	3	3	23408	23416	9
	70	A	1	8	23592	23599	8
	71	T	1	10	27457	27466	10
	72	AT	2	6	28360	28371	12
	73	A	1	8	28372	28379	8
	74	T	1	8	29288	29295	8
	75	CTTG	4	3	29465	29476	12
	76	T	1	9	31455	31463	9
	77	T	1	11	31693	31703	11
	78	A	1	9	32006	32014	9
	79	CT	2	4	32026	32033	8
	80	A	1	17	32414	32430	17
	81	A	1	8	32813	32820	8
	82	ATTA	4	3	33755	33766	12
	83	T	1	8	34054	34061	8
	84	T	1	9	34577	34585	9
	85	T	1	14	34765	34778	14
	86	T	1	8	35295	35302	8
	87	GA	2	4	38095	38102	8
	88	TA	2	4	38286	38293	8
	89	A	1	9	38820	38828	9
	90	G	1	9	38833	38841	9
	91	AAT	3	3	38890	38898	9
	92	A	1	13	38943	38955	13
	93	AT	2	4	39037	39044	8
	94	TA	2	5	39049	39058	10
	95	A	1	8	39564	39571	8
	96	C	1	8	39713	39720	8
	97	TAA	3	3	39734	39742	9
	98	TCT	3	3	40314	40322	9
	99	ATG	3	3	44076	44084	9
	100	ATT	3	3	45338	45346	9
	101	AT	2	4	45450	45457	8
	102	A	1	8	45642	45649	8
	103	T	1	8	45903	45910	8
	104	T	1	9	45987	45995	9

Species	Id	Motif	Type	Repeat	Start	End	Length
	105	TAAG	4	3	46244	46255	12
	106	T	1	11	47361	47371	11
	107	T	1	10	47399	47408	10
	108	A	1	9	47463	47471	9
	109	A	1	8	47506	47513	8
	110	A	1	11	47759	47769	11
	111	A	1	8	47775	47782	8
	112	A	1	9	48103	48111	9
	113	A	1	9	48260	48268	9
	114	A	1	8	48318	48325	8
	115	T	1	9	48384	48392	9
	116	T	1	8	49850	49857	8
	117	T	1	8	49859	49866	8
	118	AT	2	4	50162	50169	8
	119	A	1	9	50477	50485	9
	120	AT	2	5	50541	50550	10
	121	AT	2	4	50555	50562	8
	122	T	1	15	51392	51406	15
	123	T	1	8	53628	53635	8
	124	T	1	9	54230	54238	9
	125	T	1	10	54369	54378	10
	126	T	1	8	54433	54440	8
	127	T	1	10	54608	54617	10
	128	A	1	10	55613	55622	10
	129	T	1	10	57524	57533	10
	130	ATA	3	3	57578	57586	9
	131	A	1	11	57629	57639	11
	132	T	1	8	57982	57989	8
	133	T	1	11	58000	58010	11
	134	TTG	3	3	58309	58317	9
	135	AT	2	4	58642	58649	8
	136	GA	2	4	58826	58833	8
	137	GCT	3	3	59720	59728	9
	138	A	1	9	59929	59937	9
	139	AT	2	4	60284	60291	8
	140	AT	2	4	60749	60756	8
	141	AGT	3	3	61006	61014	9
	142	GGA	3	3	61368	61376	9
	143	A	1	11	62217	62227	11
	144	A	1	8	62471	62478	8
	145	AT	2	4	62605	62612	8
	146	T	1	8	62855	62862	8
	147	T	1	8	63406	63413	8
	148	GAA	3	3	63803	63811	9
	149	TCTT	4	3	63841	63852	12
	150	TC	2	4	64125	64132	8

Species	Id	Motif	Type	Repeat	Start	End	Length
	151	T	1	8	64157	64164	8
	152	TC	2	4	64306	64313	8
	153	T	1	9	64350	64358	9
	154	TC	2	5	64795	64804	10
	155	AT	2	4	65709	65716	8
	156	C	1	8	65995	66002	8
	157	A	1	8	66069	66076	8
	158	CTT	3	3	66982	66990	9
	159	AT	2	4	67314	67321	8
	160	T	1	11	67467	67477	11
	161	TTA	3	3	67598	67606	9
	162	TTA	3	3	67843	67851	9
	163	T	1	10	68790	68799	10
	164	A	1	10	68864	68873	10
	165	A	1	8	68883	68890	8
	166	A	1	8	69290	69297	8
	167	ATT	3	3	69349	69357	9
	168	T	1	11	70095	70105	11
	169	T	1	8	70603	70610	8
	170	ATA	3	3	70626	70634	9
	171	TA	2	4	70642	70649	8
	172	AT	2	4	70832	70839	8
	173	A	1	8	70896	70903	8
	174	T	1	8	71051	71058	8
	175	T	1	11	71143	71153	11
	176	TA	2	4	71727	71734	8
	177	A	1	8	71778	71785	8
	178	AAC	3	3	72034	72042	9
	179	ATAA	4	3	72159	72170	12
	180	A	1	8	72274	72281	8
	181	T	1	9	73439	73447	9
	182	T	1	9	73849	73857	9
	183	T	1	11	74402	74412	11
	184	A	1	11	74584	74594	11
	185	T	1	11	74668	74678	11
	186	A	1	10	75069	75078	10
	187	T	1	9	75109	75117	9
	188	T	1	9	75169	75177	9
	189	A	1	13	75538	75550	13
	190	A	1	8	76204	76211	8
	191	T	1	8	77070	77077	8
	192	TTG	3	3	77085	77093	9
	193	TCT	3	3	77718	77726	9
	194	TTTC	4	3	78708	78719	12
	195	T	1	8	78971	78978	8
	196	TTC	3	3	82067	82075	9

Species	Id	Motif	Type	Repeat	Start	End	Length
	197	ATA	3	3	82770	82778	9
	198	T	1	13	84289	84301	13
	199	A	1	10	84835	84844	10
	200	T	1	8	85520	85527	8
	201	TTTC	4	3	86073	86084	12
	202	T	1	12	86136	86147	12
	203	T	1	10	86392	86401	10
	204	T	1	9	86432	86440	9
	205	T	1	8	86447	86454	8
	206	T	1	10	87195	87204	10
	207	TGC	3	3	87427	87435	9
	208	T	1	9	87960	87968	9
	209	T	1	8	87998	88005	8
	210	CTT	3	3	88094	88102	9
	211	TA	2	4	88586	88593	8
	212	GA	2	4	90203	90210	8
	213	GA	2	4	90215	90222	8
	214	GA	2	4	91202	91209	8
	215	ATT	3	3	92969	92977	9
	216	A	1	9	93384	93392	9
	217	GA	2	4	93405	93412	8
	218	TCT	3	3	94088	94096	9
	219	CTT	3	3	94194	94202	9
	220	GGT	3	3	94458	94466	9
	221	GAA	3	3	95921	95929	9
	222	TA	2	4	96758	96765	8
	223	TA	2	4	98201	98208	8
	224	AG	2	4	98961	98968	8
	225	AGA	3	3	99256	99264	9
	226	T	1	8	99602	99609	8
	227	AGA	3	3	100696	100704	9
	228	T	1	8	102909	102916	8
	229	AAG	3	3	103959	103967	9
	230	T	1	8	106890	106897	8
	231	TTC	3	4	107218	107229	12
	232	CTG	3	3	108673	108681	9
	233	CT	2	4	110229	110236	8
	234	AT	2	4	111941	111948	8
	235	A	1	9	111957	111965	9
	236	T	1	9	112107	112115	9
	237	CAA	3	3	113727	113735	9
	238	T	1	9	114364	114372	9
	239	A	1	8	114392	114399	8
	240	A	1	8	115124	115131	8
	241	ATT	3	3	115143	115151	9
	242	TAA	3	3	116082	116090	9

Species	Id	Motif	Type	Repeat	Start	End	Length
	243	ATAG	4	3	116476	116487	12
	244	ATT	3	3	116957	116965	9
	245	A	1	10	117094	117103	10
	246	T	1	9	117114	117122	9
	247	A	1	11	117349	117359	11
	248	A	1	10	117525	117534	10
	249	A	1	10	117812	117821	10
	250	T	1	9	117837	117845	9
	251	A	1	9	117924	117932	9
	252	T	1	8	118191	118198	8
	253	A	1	10	118419	118428	10
	254	T	1	8	118768	118775	8
	255	A	1	8	119217	119224	8
	256	T	1	8	119303	119310	8
	257	A	1	10	119671	119680	10
	258	T	1	9	119858	119866	9
	259	T	1	10	119884	119893	10
	260	AAT	3	3	119918	119926	9
	261	AATA	4	3	120175	120186	12
	262	T	1	8	120566	120573	8
	263	A	1	8	121500	121507	8
	264	A	1	8	123518	123525	8
	265	T	1	12	123608	123619	12
	266	TTA	3	3	123623	123631	9
	267	AT	2	4	124921	124928	8
	268	ATA	3	3	124994	125002	9
	269	A	1	9	125140	125148	9
	270	T	1	10	125213	125222	10
	271	T	1	16	125618	125633	16
	272	A	1	8	127629	127636	8
	273	T	1	9	127956	127964	9
	274	TAA	3	3	128189	128197	9
	275	AT	2	4	128348	128355	8
	276	T	1	8	128770	128777	8
	277	T	1	8	129311	129318	8
	278	T	1	9	129542	129550	9
	279	ATC	3	3	129788	129796	9
	280	TAAT	4	3	130041	130052	12
	281	T	1	8	130546	130553	8
	282	A	1	8	130562	130569	8
	283	T	1	8	130702	130709	8
	284	T	1	9	130858	130866	9
	285	TTA	3	3	131029	131037	9
	286	A	1	10	131094	131103	10
	287	T	1	8	131161	131168	8
	288	T	1	9	131681	131689	9

Species	Id	Motif	Type	Repeat	Start	End	Length
	289	TC	2	4	132090	132097	8
	290	T	1	9	132173	132181	9
	291	T	1	8	132290	132297	8
	292	A	1	11	132448	132458	11
	293	A	1	8	132793	132800	8
	294	TTG	3	3	133488	133496	9
	295	A	1	10	135108	135117	10
	296	T	1	9	135259	135267	9
	297	AT	2	4	135276	135283	8
	298	AG	2	4	136988	136995	8
	299	CAG	3	3	138543	138551	9
	300	AGA	3	4	139994	140005	12
	301	A	1	8	140327	140334	8
	302	CTT	3	3	143257	143265	9
	303	A	1	8	144308	144315	8
	304	TTC	3	3	146519	146527	9
	305	A	1	8	147615	147622	8
	306	TCT	3	3	147960	147968	9
	307	CT	2	4	148256	148263	8
	308	AT	2	4	149015	149022	8
	309	TA	2	4	150459	150466	8
	310	TTC	3	3	151295	151303	9
	311	ACC	3	3	152758	152766	9
	312	AAG	3	3	153022	153030	9
	313	AGA	3	3	153128	153136	9
	314	TC	2	4	153812	153819	8
	315	T	1	9	153832	153840	9
	316	AAT	3	3	154247	154255	9
	317	TC	2	4	156015	156022	8
	318	TC	2	4	157002	157009	8
	319	TC	2	4	157014	157021	8
	320	AT	2	4	158630	158637	8
	321	GAA	3	3	159121	159129	9
	322	A	1	8	159219	159226	8
Plinia edulis	1	A	1	9	263	271	9
	2	AAG	3	3	782	790	9
	3	CAG	3	4	1137	1148	12
	4	A	1	8	1946	1953	8
	5	T	1	8	2122	2129	8
	6	TCC	3	3	2298	2306	9
	7	T	1	9	2433	2441	9
	8	A	1	10	3724	3733	10
	9	A	1	10	4483	4492	10
	10	A	1	13	4650	4662	13
	11	T	1	12	4703	4714	12
	12	TA	2	4	4776	4783	8

Species	Id	Motif	Type	Repeat	Start	End	Length
	13	A	1	10	4821	4830	10
	14	AGAT	4	3	4839	4850	12
	15	A	1	8	5458	5465	8
	16	A	1	10	5623	5632	10
	17	A	1	10	6501	6510	10
	18	A	1	9	6660	6668	9
	19	A	1	9	6730	6738	9
	20	T	1	11	7896	7906	11
	21	A	1	10	7977	7986	10
	22	A	1	9	8214	8222	9
	23	T	1	12	8379	8390	12
	24	T	1	9	8512	8520	9
	25	A	1	11	8522	8532	11
	26	T	1	10	8673	8682	10
	27	A	1	10	8755	8764	10
	28	A	1	10	8781	8790	10
	29	A	1	9	8953	8961	9
	30	T	1	11	9133	9143	11
	31	TAT	3	3	9336	9344	9
	32	A	1	11	9383	9393	11
	33	A	1	8	9571	9578	8
	34	T	1	9	9607	9615	9
	35	T	1	10	9924	9933	10
	36	AT	2	4	10547	10554	8
	37	A	1	9	10725	10733	9
	38	A	1	9	10741	10749	9
	39	T	1	8	10968	10975	8
	40	A	1	10	11037	11046	10
	41	A	1	10	11055	11064	10
	42	ATTA	4	3	11075	11086	12
	43	ATTT	4	3	11123	11134	12
	44	A	1	9	12689	12697	9
	45	T	1	9	13248	13256	9
	46	A	1	8	13480	13487	8
	47	TAG	3	3	13602	13610	9
	48	T	1	13	14066	14078	13
	49	TAA	3	4	14261	14272	12
	50	A	1	8	14348	14355	8
	51	A	1	8	14530	14537	8
	52	A	1	11	14822	14832	11
	53	T	1	10	15592	15601	10
	54	T	1	8	15635	15642	8
	55	ATT	3	3	15643	15651	9
	56	GGTA	4	3	15690	15701	12
	57	T	1	11	15763	15773	11
	58	AAC	3	3	16069	16077	9

Species	Id	Motif	Type	Repeat	Start	End	Length
	59	TTA	3	3	16878	16886	9
	60	T	1	11	17618	17628	11
	61	A	1	12	17629	17640	12
	62	T	1	8	17798	17805	8
	63	T	1	8	18467	18474	8
	64	T	1	11	19850	19860	11
	65	A	1	8	19993	20000	8
	66	A	1	8	22433	22440	8
	67	T	1	10	22446	22455	10
	68	TTC	3	3	23462	23470	9
	69	A	1	8	23646	23653	8
	70	T	1	10	27511	27520	10
	71	AT	2	5	28414	28423	10
	72	A	1	8	28424	28431	8
	73	CTTG	4	3	29512	29523	12
	74	C	1	8	30894	30901	8
	75	AAG	3	3	31881	31889	9
	76	A	1	9	32039	32047	9
	77	CT	2	4	32059	32066	8
	78	A	1	12	32450	32461	12
	79	A	1	8	32844	32851	8
	80	A	1	9	32916	32924	9
	81	AT	2	4	34011	34018	8
	82	GAA	3	3	34609	34617	9
	83	T	1	9	34678	34686	9
	84	T	1	10	34866	34875	10
	85	T	1	8	34882	34889	8
	86	T	1	8	38094	38101	8
	87	GA	2	4	38216	38223	8
	88	A	1	8	38954	38961	8
	89	G	1	8	38966	38973	8
	90	AAT	3	3	39022	39030	9
	91	A	1	15	39074	39088	15
	92	AT	2	4	39170	39177	8
	93	TA	2	5	39182	39191	10
	94	AT	2	4	39194	39201	8
	95	TAA	3	3	39460	39468	9
	96	A	1	8	39697	39704	8
	97	TAA	3	3	39866	39874	9
	98	TCT	3	3	40443	40451	9
	99	ATG	3	3	41981	41989	9
	100	ATG	3	3	44205	44213	9
	101	ATT	3	3	45467	45475	9
	102	AT	2	4	45580	45587	8
	103	A	1	8	45738	45745	8
	104	A	1	8	45763	45770	8

Species	Id	Motif	Type	Repeat	Start	End	Length
	105	T	1	9	46024	46032	9
	106	T	1	9	46109	46117	9
	107	T	1	13	46242	46254	13
	108	TAAG	4	3	46372	46383	12
	109	T	1	10	47489	47498	10
	110	T	1	11	47526	47536	11
	111	A	1	10	47591	47600	10
	112	A	1	8	47629	47636	8
	113	A	1	10	47882	47891	10
	114	A	1	8	47897	47904	8
	115	A	1	10	48225	48234	10
	116	A	1	12	48382	48393	12
	117	A	1	9	48444	48452	9
	118	T	1	9	48511	48519	9
	119	T	1	10	50057	50066	10
	120	T	1	9	50091	50099	9
	121	T	1	8	50101	50108	8
	122	TA	2	4	50368	50375	8
	123	AT	2	4	50380	50387	8
	124	AT	2	4	50773	50780	8
	125	T	1	10	51615	51624	10
	126	T	1	15	53888	53902	15
	127	T	1	9	54496	54504	9
	128	T	1	10	54635	54644	10
	129	T	1	10	54895	54904	10
	130	A	1	9	55900	55908	9
	131	T	1	10	57810	57819	10
	132	ATA	3	3	57864	57872	9
	133	A	1	8	57916	57923	8
	134	T	1	10	58285	58294	10
	135	A	1	9	58399	58407	9
	136	TTG	3	3	58594	58602	9
	137	AT	2	4	58927	58934	8
	138	GA	2	4	59111	59118	8
	139	GCT	3	3	60005	60013	9
	140	A	1	8	60198	60205	8
	141	T	1	8	60450	60457	8
	142	A	1	8	60510	60517	8
	143	AT	2	4	60554	60561	8
	144	AT	2	4	61019	61026	8
	145	AGT	3	3	61276	61284	9
	146	GGA	3	3	61638	61646	9
	147	A	1	8	62489	62496	8
	148	TA	2	4	62580	62587	8
	149	A	1	8	62742	62749	8
	150	AT	2	4	62891	62898	8

Species	Id	Motif	Type	Repeat	Start	End	Length
	151	T	1	8	63141	63148	8
	152	T	1	8	63692	63699	8
	153	GAA	3	3	64089	64097	9
	154	TCTT	4	3	64127	64138	12
	155	TC	2	4	64426	64433	8
	156	T	1	8	64458	64465	8
	157	TC	2	4	64612	64619	8
	158	T	1	9	64656	64664	9
	159	TC	2	5	65101	65110	10
	160	CTA	3	3	65822	65830	9
	161	AT	2	4	66011	66018	8
	162	C	1	8	66297	66304	8
	163	A	1	8	66371	66378	8
	164	CTT	3	3	67284	67292	9
	165	AT	2	4	67599	67606	8
	166	T	1	9	67752	67760	9
	167	TTA	3	3	67883	67891	9
	168	TTA	3	3	68128	68136	9
	169	T	1	11	69084	69094	11
	170	A	1	8	69154	69161	8
	171	A	1	9	69171	69179	9
	172	A	1	9	69579	69587	9
	173	T	1	10	70340	70349	10
	174	A	1	10	70538	70547	10
	175	T	1	8	70862	70869	8
	176	ATA	3	3	70885	70893	9
	177	AT	2	4	71078	71085	8
	178	T	1	8	71296	71303	8
	179	T	1	9	71383	71391	9
	180	T	1	10	71644	71653	10
	181	AAC	3	3	72280	72288	9
	182	ATAA	4	3	72405	72416	12
	183	A	1	8	72514	72521	8
	184	T	1	9	73669	73677	9
	185	T	1	8	74079	74086	8
	186	T	1	14	74631	74644	14
	187	A	1	9	74818	74826	9
	188	T	1	10	74900	74909	10
	189	A	1	9	75300	75308	9
	190	CT	2	4	75332	75339	8
	191	T	1	12	75340	75351	12
	192	T	1	10	75403	75412	10
	193	A	1	18	75780	75797	18
	194	T	1	12	76426	76437	12
	195	T	1	8	77298	77305	8
	196	TTG	3	3	77313	77321	9

Species	Id	Motif	Type	Repeat	Start	End	Length
	197	TCT	3	3	77946	77954	9
	198	TTTC	4	4	78936	78951	16
	199	T	1	8	79203	79210	8
	200	T	1	8	81989	81996	8
	201	TTC	3	3	82333	82341	9
	202	ATA	3	3	83036	83044	9
	203	T	1	8	84554	84561	8
	204	A	1	9	85094	85102	9
	205	TTTC	4	3	86333	86344	12
	206	T	1	10	86395	86404	10
	207	T	1	19	86649	86667	19
	208	T	1	8	86694	86701	8
	209	T	1	9	86708	86716	9
	210	T	1	10	87457	87466	10
	211	TGC	3	3	87677	87685	9
	212	AT	2	4	87814	87821	8
	213	T	1	9	88204	88212	9
	214	T	1	8	88242	88249	8
	215	CTT	3	3	88338	88346	9
	216	TA	2	4	88830	88837	8
	217	GA	2	4	90447	90454	8
	218	GA	2	4	90459	90466	8
	219	GA	2	4	91446	91453	8
	220	ATT	3	3	93213	93221	9
	221	A	1	9	93628	93636	9
	222	GA	2	4	93649	93656	8
	223	TCT	3	3	94332	94340	9
	224	CTT	3	3	94438	94446	9
	225	GGT	3	3	94702	94710	9
	226	GAA	3	3	96183	96191	9
	227	TA	2	4	97020	97027	8
	228	TA	2	4	98463	98470	8
	229	AG	2	4	99223	99230	8
	230	AGA	3	3	99518	99526	9
	231	T	1	8	99864	99871	8
	232	AGA	3	3	100958	100966	9
	233	T	1	8	103173	103180	8
	234	AAG	3	3	104233	104241	9
	235	T	1	8	107164	107171	8
	236	TTC	3	4	107492	107503	12
	237	CTG	3	3	108947	108955	9
	238	CT	2	4	110508	110515	8
	239	A	1	10	112236	112245	10
	240	T	1	10	112387	112396	10
	241	CAA	3	3	114008	114016	9
	242	ATT	3	3	114640	114648	9

Species	Id	Motif	Type	Repeat	Start	End	Length
	243	T	1	10	114702	114711	10
	244	A	1	8	114731	114738	8
	245	A	1	8	115463	115470	8
	246	ATT	3	3	115482	115490	9
	247	T	1	8	115525	115532	8
	248	TAA	3	3	116421	116429	9
	249	ATAG	4	3	116815	116826	12
	250	ATT	3	3	117296	117304	9
	251	A	1	9	117433	117441	9
	252	A	1	9	117674	117682	9
	253	A	1	8	117846	117853	8
	254	A	1	10	118130	118139	10
	255	A	1	10	118141	118150	10
	256	T	1	11	118166	118176	11
	257	T	1	8	118521	118528	8
	258	A	1	11	118749	118759	11
	259	AAT	3	3	118798	118806	9
	260	TAT	3	4	118808	118819	12
	261	T	1	8	119092	119099	8
	262	A	1	8	119541	119548	8
	263	T	1	8	119627	119634	8
	264	A	1	10	119995	120004	10
	265	T	1	10	120182	120191	10
	266	T	1	8	120209	120216	8
	267	AAT	3	3	120241	120249	9
	268	AATA	4	3	120476	120487	12
	269	T	1	8	120867	120874	8
	270	A	1	8	121801	121808	8
	271	A	1	8	121883	121890	8
	272	TA	2	4	123534	123541	8
	273	T	1	9	123606	123614	9
	274	A	1	8	123851	123858	8
	275	T	1	10	123941	123950	10
	276	AT	2	4	125247	125254	8
	277	ATA	3	3	125320	125328	9
	278	A	1	9	125466	125474	9
	279	T	1	9	125539	125547	9
	280	T	1	10	125942	125951	10
	281	A	1	8	127944	127951	8
	282	T	1	8	128271	128278	8
	283	TAA	3	3	128499	128507	9
	284	AT	2	4	128658	128665	8
	285	T	1	8	129080	129087	8
	286	T	1	8	129621	129628	8
	287	T	1	9	129852	129860	9
	288	ATC	3	3	130086	130094	9

Species	Id	Motif	Type	Repeat	Start	End	Length
	289	TAAT	4	3	130339	130350	12
	290	T	1	8	130844	130851	8
	291	A	1	8	130860	130867	8
	292	T	1	9	131156	131164	9
	293	TTA	3	3	131327	131335	9
	294	A	1	11	131391	131401	11
	295	T	1	8	131459	131466	8
	296	T	1	9	131979	131987	9
	297	TC	2	4	132385	132392	8
	298	T	1	8	132468	132475	8
	299	T	1	8	132585	132592	8
	300	A	1	10	132743	132752	10
	301	A	1	8	133088	133095	8
	302	TTG	3	3	133804	133812	9
	303	A	1	10	135424	135433	10
	304	T	1	10	135575	135584	10
	305	AG	2	4	137305	137312	8
	306	CAG	3	3	138865	138873	9
	307	AGA	3	4	140316	140327	12
	308	A	1	8	140649	140656	8
	309	CTT	3	3	143579	143587	9
	310	A	1	8	144640	144647	8
	311	TTC	3	3	146853	146861	9
	312	A	1	8	147949	147956	8
	313	TCT	3	3	148294	148302	9
	314	CT	2	4	148590	148597	8
	315	AT	2	4	149349	149356	8
	316	TA	2	4	150793	150800	8
	317	TTC	3	3	151629	151637	9
	318	ACC	3	3	153110	153118	9
	319	AAG	3	3	153374	153382	9
	320	AGA	3	3	153480	153488	9
	321	TC	2	4	154164	154171	8
	322	T	1	9	154184	154192	9
	323	AAT	3	3	154599	154607	9
	324	TC	2	4	156367	156374	8
	325	TC	2	4	157354	157361	8
	326	TC	2	4	157366	157373	8
	327	AT	2	4	158982	158989	8
	328	GAA	3	3	159473	159481	9
	329	A	1	8	159571	159578	8
	330	A	1	9	159608	159616	9
Psidium cattleianum	1	A	1	9	278	286	9
	2	CAG	3	4	1151	1162	12
	3	A	1	8	1958	1965	8
	4	TCC	3	3	2309	2317	9

Species	Id	Motif	Type	Repeat	Start	End	Length
	5	T	1	9	2444	2452	9
	6	A	1	11	3735	3745	11
	7	A	1	9	3965	3973	9
	8	T	1	8	4254	4261	8
	9	A	1	8	4490	4497	8
	10	A	1	12	4663	4674	12
	11	T	1	9	4716	4724	9
	12	A	1	8	4729	4736	8
	13	T	1	8	4785	4792	8
	14	A	1	8	4922	4929	8
	15	A	1	9	5464	5472	9
	16	A	1	8	5623	5630	8
	17	A	1	8	6501	6508	8
	18	A	1	8	6658	6665	8
	19	A	1	8	6727	6734	8
	20	TA	2	4	7521	7528	8
	21	T	1	10	7875	7884	10
	22	A	1	8	7947	7954	8
	23	A	1	9	8182	8190	9
	24	T	1	8	8348	8355	8
	25	T	1	10	8477	8486	10
	26	A	1	11	8488	8498	11
	27	A	1	11	8739	8749	11
	28	A	1	9	8909	8917	9
	29	A	1	10	9038	9047	10
	30	T	1	11	9075	9085	11
	31	TAT	3	3	9282	9290	9
	32	A	1	9	9330	9338	9
	33	T	1	9	9552	9560	9
	34	T	1	9	9869	9877	9
	35	AT	2	4	10494	10501	8
	36	A	1	9	10689	10697	9
	37	T	1	8	10909	10916	8
	38	A	1	8	10981	10988	8
	39	ATTA	4	3	10999	11010	12
	40	ATTT	4	3	11047	11058	12
	41	A	1	8	12613	12620	8
	42	T	1	9	13171	13179	9
	43	A	1	8	13403	13410	8
	44	TAG	3	3	13525	13533	9
	45	T	1	10	13989	13998	10
	46	A	1	8	14269	14276	8
	47	A	1	10	14742	14751	10
	48	T	1	9	15473	15481	9
	49	TA	2	4	15498	15505	8
	50	T	1	8	15510	15517	8

Species	Id	Motif	Type	Repeat	Start	End	Length
	51	A	1	8	15533	15540	8
	52	T	1	9	15634	15642	9
	53	AAC	3	3	15938	15946	9
	54	TTA	3	3	16753	16761	9
	55	A	1	8	17498	17505	8
	56	T	1	9	17663	17671	9
	57	T	1	11	19716	19726	11
	58	A	1	8	19859	19866	8
	59	A	1	8	22299	22306	8
	60	T	1	10	22312	22321	10
	61	TTC	3	3	23328	23336	9
	62	A	1	8	23512	23519	8
	63	T	1	10	27377	27386	10
	64	AT	2	4	28280	28287	8
	65	A	1	11	28288	28298	11
	66	T	1	8	29211	29218	8
	67	CTTG	4	3	29388	29399	12
	68	T	1	11	31587	31597	11
	69	A	1	9	31889	31897	9
	70	CT	2	4	31909	31916	8
	71	A	1	10	32237	32246	10
	72	A	1	8	32629	32636	8
	73	A	1	10	32701	32710	10
	74	AATGG	5	3	33175	33189	15
	75	ATTA	4	3	33622	33633	12
	76	T	1	8	33930	33937	8
	77	T	1	9	34453	34461	9
	78	T	1	10	34641	34650	10
	79	T	1	9	34657	34665	9
	80	T	1	8	35162	35169	8
	81	T	1	9	37855	37863	9
	82	GA	2	4	37967	37974	8
	83	TA	2	4	38158	38165	8
	84	A	1	8	38706	38713	8
	85	G	1	9	38718	38726	9
	86	AAT	3	3	38790	38798	9
	87	A	1	9	38833	38841	9
	88	AT	2	4	38924	38931	8
	89	TA	2	5	38936	38945	10
	90	TAA	3	3	39198	39206	9
	91	A	1	9	39236	39244	9
	92	A	1	9	39450	39458	9
	93	TAA	3	3	39619	39627	9
	94	TCT	3	3	40198	40206	9
	95	ATG	3	3	41736	41744	9
	96	ATG	3	3	43960	43968	9

Species	Id	Motif	Type	Repeat	Start	End	Length
	97	AT	2	4	45334	45341	8
	98	A	1	8	45494	45501	8
	99	A	1	8	45519	45526	8
	100	T	1	8	45780	45787	8
	101	A	1	8	45848	45855	8
	102	T	1	8	45864	45871	8
	103	TAAG	4	3	46119	46130	12
	104	T	1	10	47235	47244	10
	105	T	1	12	47272	47283	12
	106	A	1	8	47373	47380	8
	107	A	1	11	47626	47636	11
	108	A	1	8	47642	47649	8
	109	A	1	9	47970	47978	9
	110	A	1	10	48127	48136	10
	111	A	1	9	48186	48194	9
	112	T	1	9	48253	48261	9
	113	T	1	10	49827	49836	10
	114	T	1	8	49838	49845	8
	115	AT	2	5	50133	50142	10
	116	A	1	10	50450	50459	10
	117	AT	2	5	50515	50524	10
	118	T	1	10	51355	51364	10
	119	T	1	9	53627	53635	9
	120	T	1	9	54183	54191	9
	121	T	1	8	54322	54329	8
	122	T	1	9	54580	54588	9
	123	A	1	9	55584	55592	9
	124	T	1	10	57495	57504	10
	125	ATA	3	3	57549	57557	9
	126	A	1	8	57602	57609	8
	127	T	1	8	57952	57959	8
	128	T	1	10	57970	57979	10
	129	A	1	8	58084	58091	8
	130	TTG	3	3	58278	58286	9
	131	AT	2	4	58611	58618	8
	132	GA	2	4	58795	58802	8
	133	GCT	3	3	59689	59697	9
	134	A	1	8	59884	59891	8
	135	T	1	8	60133	60140	8
	136	AT	2	4	60236	60243	8
	137	AT	2	4	60701	60708	8
	138	AGT	3	3	60952	60960	9
	139	GGA	3	3	61314	61322	9
	140	A	1	8	62153	62160	8
	141	TA	2	4	62244	62251	8
	142	A	1	8	62406	62413	8

Species	Id	Motif	Type	Repeat	Start	End	Length
	143	AT	2	6	62535	62546	12
	144	T	1	8	62789	62796	8
	145	T	1	8	63340	63347	8
	146	GAA	3	3	63737	63745	9
	147	TCTT	4	3	63775	63786	12
	148	T	1	8	64101	64108	8
	149	T	1	9	64292	64300	9
	150	TC	2	5	64739	64748	10
	151	CTA	3	3	65460	65468	9
	152	C	1	8	65935	65942	8
	153	A	1	8	66009	66016	8
	154	CTT	3	3	66922	66930	9
	155	T	1	8	67033	67040	8
	156	AT	2	4	67243	67250	8
	157	T	1	10	67396	67405	10
	158	TTA	3	3	67526	67534	9
	159	ATT	3	5	67767	67781	15
	160	T	1	8	68727	68734	8
	161	A	1	9	68799	68807	9
	162	A	1	8	68817	68824	8
	163	A	1	9	69223	69231	9
	164	T	1	10	69975	69984	10
	165	T	1	8	70494	70501	8
	166	AT	2	4	70726	70733	8
	167	T	1	8	70932	70939	8
	168	T	1	10	71024	71033	10
	169	T	1	8	71286	71293	8
	170	TA	2	4	71606	71613	8
	171	A	1	8	71657	71664	8
	172	AAC	3	3	71913	71921	9
	173	ATAA	4	3	72038	72049	12
	174	A	1	8	72052	72059	8
	175	A	1	9	72150	72158	9
	176	T	1	11	73305	73315	11
	177	T	1	9	73716	73724	9
	178	T	1	12	74270	74281	12
	179	A	1	9	74454	74462	9
	180	T	1	13	74536	74548	13
	181	T	1	12	74973	74984	12
	182	T	1	8	75036	75043	8
	183	A	1	9	75409	75417	9
	184	A	1	8	76075	76082	8
	185	T	1	8	76941	76948	8
	186	TTG	3	3	76956	76964	9
	187	TCT	3	3	77589	77597	9
	188	TTTC	4	3	78594	78605	12

Species	Id	Motif	Type	Repeat	Start	End	Length
	189	T	1	8	78857	78864	8
	190	TTC	3	3	80809	80817	9
	191	T	1	8	81627	81634	8
	192	TTC	3	3	81971	81979	9
	193	T	1	8	82468	82475	8
	194	ATA	3	3	82674	82682	9
	195	T	1	9	84193	84201	9
	196	A	1	10	84735	84744	10
	197	TTTC	4	3	85950	85961	12
	198	T	1	9	86012	86020	9
	199	T	1	11	86265	86275	11
	200	T	1	10	86319	86328	10
	201	T	1	10	87069	87078	10
	202	TGC	3	3	87286	87294	9
	203	T	1	9	87803	87811	9
	204	T	1	8	87841	87848	8
	205	CTT	3	3	87937	87945	9
	206	TA	2	4	88429	88436	8
	207	GA	2	4	90046	90053	8
	208	GA	2	4	90058	90065	8
	209	GA	2	4	91045	91052	8
	210	ATT	3	3	92812	92820	9
	211	A	1	9	93227	93235	9
	212	GA	2	4	93248	93255	8
	213	TCT	3	3	93931	93939	9
	214	CTT	3	3	94037	94045	9
	215	GGT	3	3	94301	94309	9
	216	GAA	3	3	95782	95790	9
	217	TA	2	4	96619	96626	8
	218	TA	2	4	98054	98061	8
	219	AG	2	4	98814	98821	8
	220	AGA	3	3	99109	99117	9
	221	T	1	8	99455	99462	8
	222	AGA	3	3	100549	100557	9
	223	T	1	8	102764	102771	8
	224	AAG	3	3	103818	103826	9
	225	T	1	8	106749	106756	8
	226	TTC	3	4	107077	107088	12
	227	CTG	3	3	108532	108540	9
	228	CT	2	4	110093	110100	8
	229	AT	2	4	111805	111812	8
	230	A	1	9	111821	111829	9
	231	T	1	10	111971	111980	10
	232	CAA	3	3	113592	113600	9
	233	TTAT	4	3	114204	114215	12
	234	T	1	10	114271	114280	10

Species	Id	Motif	Type	Repeat	Start	End	Length
	235	T	1	8	114282	114289	8
	236	A	1	8	114309	114316	8
	237	A	1	8	114689	114696	8
	238	A	1	8	115041	115048	8
	239	ATT	3	3	115060	115068	9
	240	T	1	8	115088	115095	8
	241	TAA	3	3	115984	115992	9
	242	ATAG	4	3	116378	116389	12
	243	ATT	3	3	116860	116868	9
	244	A	1	9	116988	116996	9
	245	T	1	9	117007	117015	9
	246	A	1	9	117246	117254	9
	247	A	1	9	117418	117426	9
	248	A	1	8	117710	117717	8
	249	A	1	9	117719	117727	9
	250	T	1	9	117743	117751	9
	251	A	1	9	117830	117838	9
	252	T	1	8	118097	118104	8
	253	A	1	10	118325	118334	10
	254	AAT	3	3	118373	118381	9
	255	TAT	3	3	118383	118391	9
	256	T	1	8	118671	118678	8
	257	A	1	8	119120	119127	8
	258	T	1	8	119206	119213	8
	259	A	1	9	119574	119582	9
	260	T	1	9	119784	119792	9
	261	AAT	3	3	119817	119825	9
	262	AATA	4	3	120065	120076	12
	263	T	1	8	120456	120463	8
	264	A	1	8	121461	121468	8
	265	A	1	8	123409	123416	8
	266	T	1	10	123499	123508	10
	267	AT	2	4	124805	124812	8
	268	ATA	3	3	124878	124886	9
	269	A	1	9	125024	125032	9
	270	T	1	10	125097	125106	10
	271	T	1	10	125501	125510	10
	272	T	1	8	127828	127835	8
	273	TAA	3	3	128044	128052	9
	274	AT	2	4	128204	128211	8
	275	T	1	8	128617	128624	8
	276	T	1	8	129158	129165	8
	277	T	1	9	129389	129397	9
	278	ATC	3	3	129635	129643	9
	279	TAAT	4	3	129837	129848	12
	280	T	1	9	130284	130292	9

Species	Id	Motif	Type	Repeat	Start	End	Length
	281	T	1	9	130654	130662	9
	282	TTA	3	3	130825	130833	9
	283	A	1	10	130889	130898	10
	284	T	1	8	130957	130964	8
	285	T	1	9	131477	131485	9
	286	TC	2	4	131886	131893	8
	287	T	1	9	131969	131977	9
	288	T	1	8	132086	132093	8
	289	A	1	10	132244	132253	10
	290	T	1	10	132420	132429	10
	291	A	1	8	132583	132590	8
	292	TTG	3	3	133278	133286	9
	293	A	1	11	134898	134908	11
	294	T	1	9	135050	135058	9
	295	AT	2	4	135067	135074	8
	296	AG	2	4	136779	136786	8
	297	CAG	3	3	138339	138347	9
	298	AGA	3	4	139790	139801	12
	299	A	1	8	140123	140130	8
	300	CTT	3	3	143053	143061	9
	301	A	1	8	144108	144115	8
	302	TTC	3	3	146321	146329	9
	303	A	1	8	147417	147424	8
	304	TCT	3	3	147762	147770	9
	305	CT	2	4	148058	148065	8
	306	AT	2	4	148817	148824	8
	307	TA	2	4	150253	150260	8
	308	TTC	3	3	151089	151097	9
	309	ACC	3	3	152570	152578	9
	310	AAG	3	3	152834	152842	9
	311	AGA	3	3	152940	152948	9
	312	TC	2	4	153624	153631	8
	313	T	1	9	153644	153652	9
	314	AAT	3	3	154059	154067	9
	315	TC	2	4	155827	155834	8
	316	TC	2	4	156814	156821	8
	317	TC	2	4	156826	156833	8
	318	AT	2	4	158442	158449	8
	319	GAA	3	3	158933	158941	9
	320	A	1	8	159031	159038	8
	321	A	1	9	159068	159076	9
